# Supplementary material for: Unbiased and efficient log-likelihood estimation with inverse binomial sampling
Source: PLoS Comput Biol. 2020 Dec 23;16(12):e1008483. doi: 10.1371/journal.pcbi.1008483 (PMC7758077; doi:10.1371/journal.pcbi.1008483)
Supplement: S1 Appendix — A. Further theoretical analyses: Why inverse binomial sampling works; Analysis of bias of fixed sampling; Derivation of IBS variance; Estimator variance and information inequality; A Bayesian derivation of the IBS estimator; Estimator RMSE. B. Experimental details: Orientation discrimination; Change localization; Four-in-a-row game. C. Improvements of IBS and further applications: Early stopping threshold; Reducing variance by trial-dependent repeated sampling; Bayesian inference with IBS. (PDF) [file pcbi.1008483.s001.pdf]

Unbiased and efficient log-likelihood estimation with inverse binomial sampling

Appendix S1
Supplemental methods

Bas van Opheusden*, Luigi Acerbi*, Wei Ji Ma

* These authors contributed equally to this work.
Contacts: svo@princeton.edu; luigi.acerbi@helsinki.fi

Contents

A Further theoretical analyses **1**
- A.1 Why inverse binomial sampling works 1
- A.2 Analysis of bias of fixed sampling 2
- A.3 Derivation of IBS variance 3
- A.4 Estimator variance and information inequality 5
- A.5 A Bayesian derivation of the IBS estimator 6
- A.6 Estimator RMSE 7

B Experimental details **7**
- B.1 Orientation discrimination 8
- B.2 Change localization 9
- B.3 Four-in-a-row game 12

C Improvements of IBS and further applications **14**
- C.1 Early stopping threshold 14
- C.2 Reducing variance by trial-dependent repeated sampling 17
- C.3 Bayesian inference with IBS 18

Supplemental References **20**

A Further theoretical analyses

A.1 Why inverse binomial sampling works

We start by showing that the inverse binomial sampling policy described in the Methods section of the main text, combined with the estimator $\hat{\mathcal{L}}_{\text{ibs}}$ (Eq 14 in the main text), yields a uniformly unbiased estimate of $\log p$. This

derivation follows from [1, Theorem 4.1], adapted to our special case of estimating $\log p$ instead of an arbitrary function $f(p)$:

$$\begin{aligned}\mathbb{E}\left[\hat{\mathcal{L}}_{\text{ibs}}\right] &= -\,\mathbb{E}\left[\sum_{k=1}^{K-1}\frac{1}{k}\right] = -\mathbb{E}\left[\sum_{k=1}^{\infty}\frac{1}{k}\mathbb{1}_{k<K}\right] \\ &= -\sum_{k=1}^{\infty}\frac{1}{k}\mathbb{E}\left[\mathbb{1}_{k<K}\right] = -\sum_{k=1}^{\infty}\frac{1}{k}\Pr\left(k<K\right) \\ &= -\sum_{k=1}^{\infty}\frac{1}{k}(1-p)^k = \log p.\end{aligned} \tag{S1}$$

The first equality is the definition of $\hat{\mathcal{L}}_{\text{ibs}}$ (Eq 14 in the main text), using the notational convention that $\sum_{k=1}^{0}=0$. In the second equality we introduce the indicator function $\mathbb{1}_{k<K}$ which is 1 when $k<K$ and 0 otherwise. The third equality follows by linearity of the expectation and the fourth directly from the definition of the indicator function. The fifth and second-to last equality uses the formula for the cumulative distribution function of a geometric variable, that is $\Pr(K\le k)=1-(1-p)^k$, and thus $\Pr(k<K)=(1-p)^k$. The final equality is the definition of the Taylor series of $\log p$ expanded around $p=1$. Note that this series converges for all $p\in(0,1]$.

In the derivation above, we can replace $\frac{1}{k}$ by an arbitrary set of coefficients a_k and show that

$$\mathbb{E}\left[\sum_{k=1}^{K-1}a_k\right]=\sum_{k=1}^{\infty}a_k(1-p)^k, \tag{S2}$$

for all p for which the resulting Taylor series converges. Eq S2 immediately proves two corollaries. First, we can use the inverse binomial sampling policy to estimate any analytic function of p. Second, since we can rewrite any estimator $\hat{\mathcal{L}}(K)$ as $\sum_{k=1}^{K-1}a_k$, and since Taylor series are unique, $a_k=\frac{1}{k}$ is the only choice for which $\mathbb{E}\left[\hat{\mathcal{L}}(K)\right]$ equals $\log p$. In other words, $\hat{\mathcal{L}}_{\text{ibs}}$ is the only uniformly unbiased estimator of $\log p$ with the inverse sampling policy. Therefore, it trivially is the *uniformly minimum-variance unbiased estimator* under this policy, since no other unbiased estimator exist.

A.2 Analysis of bias of fixed sampling

We provide here a more formal analysis of the bias of fixed sampling. We initially consider the estimator $\hat{\mathcal{L}}_{\text{fixed}}$ defined by Eq 11 in the main text, but we will see that our arguments hold generally for any estimator based on a fixed sampling policy.

We showed in Fig 2 of the main text that in the regime of $p\to 0$, $M\to\infty$, while keeping $pM\to\lambda$, the bias of $\hat{\mathcal{L}}_{\text{fixed}}$ tends to a master curve. This follows since, in this limit, the binomial distribution $\text{Binom}\left(\frac{\lambda}{M},M\right)$ converges to a Poisson distribution $\text{Pois}(\lambda)$ and therefore the bias converges to

$$\begin{aligned}\text{Bias}\left[\hat{\mathcal{L}}_{\text{fixed}}|p\right] &= \mathbb{E}\left[\hat{\mathcal{L}}_{\text{fixed}}-\log\frac{\lambda}{M}\right] \\ &= \exp(-\lambda)\sum_{m=0}^{\infty}\frac{\lambda^m}{m!}\log(m+1)-\log(M+1)-\log\frac{\lambda}{M} \\ &\underset{\substack{p\to 0\\ M\to\infty\\ pM\to\lambda}}{\longrightarrow} \exp(-\lambda)\sum_{m=0}^{\infty}\frac{\lambda^m}{m!}\log(m+1)-\log\lambda,\end{aligned} \tag{S3}$$

which is the master curve in Fig 2 of the main text. In particular, the bias is close to zero for $\lambda\gg 1$ and it diverges when $\lambda\ll 1$, or equivalently, for $M\gg\frac{1}{p}$ and $M\ll\frac{1}{p}$, respectively.

This asymptotic behavior is not a coincidence. In fact, it is mathematically guaranteed since the Fisher information of $\text{Pois}(\lambda)$ equals $\frac{1}{\lambda}$ and the reparametrization identity for the Fisher information yields $\mathcal{I}_f(\log\lambda) = \lambda$ [2]. In the limit of $p \ll \frac{1}{M}$, which corresponds to $\lambda \ll 1$, this Fisher information vanishes and the outcome of fixed sampling simply provides zero information about $\log\lambda$ or $\log p$. Therefore, any estimates of $\log p$ are not informed by the data and instead are a function of the regularization chosen in the estimator $\hat{\mathcal{L}}_{\text{fixed}}$ (Eq 11 in the main text). Note that the argument above does not invoke the specific form of the estimator, and therefore holds for any choice of regularization.

We can express the problem with fixed sampling more clearly using Bayesian statistics, in a formal treatment of the 'gambling' analogy we presented in the main text. The 'correct' belief about $\log\lambda$ given the outcome of fixed sampling (m) is quantified by the posterior distribution $p(\log\lambda|m)$, which is a product of the likelihood $\Pr(m|\log\lambda)$ and a prior $p(\log\lambda)$. In the limit $\lambda \ll 1$, the Poisson distribution converges to a Kronecker delta distribution concentrated on $m = 0$. In other words, almost surely none of the samples taken by the behavioral model will match the participant's response. When $m = 0$, the likelihood equals $\exp(-\lambda)$, which is mostly flat (when considered as a function of $\log\lambda$, see Fig S1) for $\log\lambda \in [-\infty, -2]$ and therefore our posterior belief ought to be dominated by the prior $p(\log\lambda)$ and become independent of the data. Therefore, we once again conclude that in the limit $p \ll \frac{1}{M}$, the fixed sampling policy provides no information to base an estimate of $\log p$ on, and it is impossible to avoid bias.

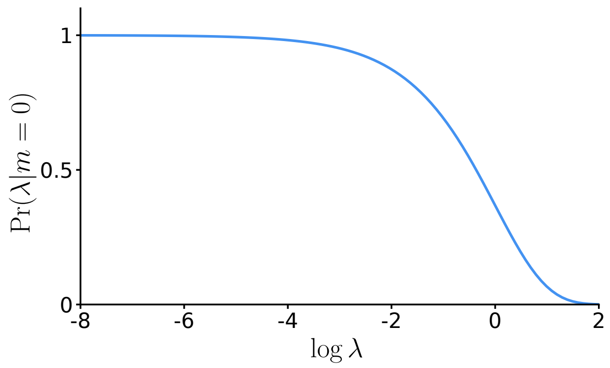

Figure S1. Likelihood function for zero 'hits'. Likelihood function of $\log\lambda$ given that fixed sampling returns $m = 0$ (none of the samples from the model match the participant's response). The likelihood is approximately flat for all $\log\lambda \leq -2$. Since λ is defined as $\frac{p}{M}$, this implies that the posterior distribution over p will be dominated by a prior rather than evidence, as quantified by the likelihood.

A.3 Derivation of IBS variance

In this section, we derive the expression for the variance of the IBS estimator (Eq 15 in the main text). We compute the variance of $\hat{L}_{\text{ibs}}$ starting from the identity

$$\text{Var}\left[\hat{L}_{\text{ibs}}\right] = \mathbb{E}\left[\left(\hat{L}_{\text{ibs}}\right)^2\right] - \left(\mathbb{E}\left[\hat{L}_{\text{ibs}}\right]\right)^2.$$

We already know the second term is equal to $(\log p)^2$, but for the purpose of this derivation, and for reasons that will become clear later, we re-write it as

$$\left(\mathbb{E}\left[\hat{L}_{\text{ibs}}\right]\right)^2 = \left(\sum_{m=1}^{\infty} \frac{1}{m}(1-p)^m\right)^2 = \sum_{m,n=1}^{\infty} \frac{1}{mn}(1-p)^{m+n}.$$

In order to write this equation as a power series in $1-p$, we collect terms with the same exponent together. Specifically, we re-index this double summation as a summation over all values of n and $m+n$ (which we label k), and substitute $k-n$ for m.

$$\sum_{m,n=1}^{\infty} \frac{1}{mn}(1-p)^{m+n} = \sum_{k=1}^{\infty}\sum_{n=1}^{k-1} \frac{1}{(k-n)n}(1-p)^{k}.$$

Note that in the second summation over n we only have to sum to $n=k-1$ since $m \equiv n-k$ has to be positive. We can carry out the internal summation over n explicitly,

$$\sum_{n=1}^{k-1} \frac{1}{(k-n)n} = \sum_{n=1}^{k-1}\left[\frac{1}{k(k-n)} + \frac{1}{kn}\right] = \frac{2}{k}\sum_{n=1}^{k-1}\frac{1}{n} = \frac{2}{k}H_{k-1}.$$

The first equality is an algebraic manipulation, the second follows by symmetry and the final equality defines H_{k-1} as the $(k-1)$-th harmonic number. Therefore, we find that

$$\left(\mathbb{E}\left[\hat{L}_{\text{ibs}}\right]\right)^2 = 2\sum_{k=1}^{\infty}\frac{H_{k-1}}{k}(1-p)^k.$$

To calculate $\mathbb{E}\left[\left(\hat{L}_{\text{ibs}}\right)^2\right]$, we use a similar rationale as Eq S1,

$$\begin{aligned}
\mathbb{E}\left[\left(\hat{L}_{\text{ibs}}\right)^2\right] &= \mathbb{E}\left[\left(-\sum_{m=1}^{K-1}\frac{1}{m}\right)^2\right] = \mathbb{E}\left[\sum_{m,n=1}^{K-1}\frac{1}{mn}\right] \\
&= \mathbb{E}\left[\sum_{m,n=1}^{\infty}\frac{1}{mn}\mathbb{1}_{m<K}\mathbb{1}_{n<K}\right] = \sum_{m,n=1}^{\infty}\frac{1}{mn}\mathbb{E}\left[\mathbb{1}_{m<K,n<K}\right] \\
&= \sum_{m,n=1}^{\infty}\frac{1}{mn}\Pr\left(\mathbb{1}_{m<K,n<K}\right) = \sum_{m,n=1}^{\infty}\frac{1}{mn}(1-p)^{\max(m,n)}.
\end{aligned}$$

In these equations, $\mathbb{1}$ again denotes an indicator function, and we use the fact that the product of indicator functions for two different events is the indicator function for the joint event. Additionally, we use that the event $m<K, n<K$ is logically equivalent to $\max(m,n)<K$. To write this double summation as a power series, we split it into three parts: one where $m<n$, one where $m=n$ and one where $m>n$. By symmetry, the first and last part are equal, and we can write

$$\mathbb{E}\left[\left(\hat{L}_{\text{ibs}}\right)^2\right] = \sum_{m=1}^{\infty}\frac{1}{mm}\left[(1-p)^{\max(m,m)}\right] + 2\sum_{m=1}^{\infty}\sum_{n=1}^{m-1}\frac{1}{mn}\left[(1-p)^{\max(m,n)}\right].$$

By re-arranging some terms, and using the fact that $\max(m,m)=m$ and $\max(m,n)=m$ for all $n<m$, we can reduce this to

$$\mathbb{E}\left[\left(\hat{L}_{\text{ibs}}\right)^2\right] = \sum_{m=1}^{\infty}\frac{1}{m^2}(1-p)^m + 2\sum_{m=1}^{\infty}\left[\sum_{n=1}^{m-1}\frac{1}{n}\right]\frac{1}{m}(1-p)^m.$$

We can now explicitly perform the summation over n in the second term and write

$$\mathbb{E}\left[\left(\hat{L}_{\text{ibs}}\right)^2\right] = \sum_{m=1}^{\infty}\frac{1}{m^2}(1-p)^m + 2\sum_{m=1}^{\infty}\frac{H_{m-1}}{m}(1-p)^m.$$

Finally, putting everything together, we obtain

$$\begin{aligned}\mathrm{Var}\left[\hat{L}_{\mathrm{ibs}}\right] &= \mathbb{E}\left[\left(\hat{L}_{\mathrm{ibs}}\right)^2\right] - \left(\mathbb{E}\left[\hat{L}_{\mathrm{ibs}}\right]\right)^2 \\ &= \sum_{m=1}^{\infty} \frac{1}{m^2}(1-p)^m + 2\sum_{m=1}^{\infty} \frac{H_{m-1}}{m}(1-p)^m - 2\sum_{k=1}^{\infty} \frac{H_{k-1}}{k}(1-p)^k \\ &= \sum_{m=1}^{\infty} \frac{1}{m^2}(1-p)^m.\end{aligned}$$

A.4 Estimator variance and information inequality

We proved in Section A.1 that $\hat{\mathcal{L}}_{\mathrm{IBS}}$ is the minimum-variance unbiased estimator of $\log p$ given the inverse binomial sampling policy. Here we show that the estimator also comes close to saturating the *information inequality*, the analogue of a Cramer-Ráo bound for an arbitrary function $f(p)$ and a non-fixed sampling policy [1],

$$\mathrm{Std}(\hat{f}|p) \geq \sqrt{\frac{p(1-p)}{\mathbb{E}\left[K|p\right])}} \left|\frac{\mathrm{d}f(p)}{\mathrm{d}p}\right|. \tag{S4}$$

In our case, where $f(p) = \log p$, the information inequality reduces to $\mathrm{Std}(\hat{\mathcal{L}}_{\mathrm{IBS}}) \geq \sqrt{1-p}$. In Fig S2, we plot the standard deviation of IBS compared to this lower bound.

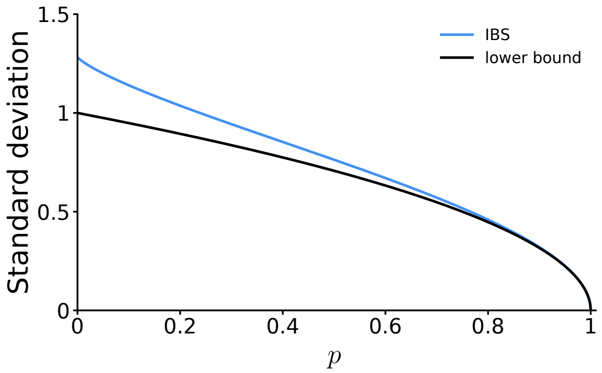

Figure S2. Information inequality. Standard deviation of IBS (Blue curve) and the lower bound given by the information inequality (black, see Eq S4). The standard deviation of IBS is within 30% of the lower bound across the entire range of p.

It may be disappointing that IBS does not match the information inequality. [3] showed that the only functions $f(p)$ for which the fixed sampling policy with M samples allows an unbiased estimator are polynomials of degree up to M, and those estimators can saturate the information equality. [4] and later [1] showed that if an unbiased estimator of a non-polynomial function $f(p)$ exists and it matches the information inequality, it must use the inverse binomial sampling policy. Moreover, de Groot derived necessary and sufficient conditions for $f(p)$ to allow such estimators [1]. Applying this argument to $f(p) = \log(p)$, the standard deviation in IBS is close (within 30%) to its theoretical minimum.

To compare the variance of IBS and fixed sampling on equal terms, we use the scaling behavior of $\hat{\mathcal{L}}_{\mathrm{fixed}}$ as $M \to \infty$. Specifically, for fixed sampling, we plot $\sqrt{M} \times \mathrm{Std}(\hat{\mathcal{L}}_{\mathrm{fixed}})$ and for IBS we plot $\frac{1}{\sqrt{p}} \times \mathrm{Std}(\hat{\mathcal{L}}_{\mathrm{IBS}})$ (see

Fig S3). With this scaling, the curves for fixed sampling again collapse onto a master curve[1]. Note that repeated-sampling IBS estimators $\hat{\mathcal{L}}_{\text{IBS-}R}$ (see Eq 17 in the main text), obtained by averaging multiple IBS estimates, overlap with the curve for regular IBS for any R.

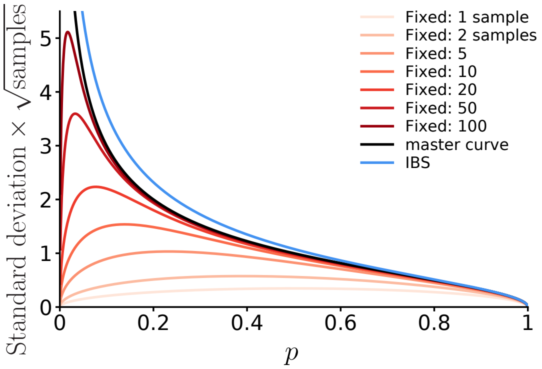

Figure S3. Comparison of variability of IBS and fixed sampling. Standard deviation times square root of the expected number of samples drawn by IBS (blue) and fixed sampling (red), and the master curve (black) that fixed sampling converges to when $M \to \infty$.

All these curves increase and diverge as $p \to 0$, reflecting the fact that estimating log-likelihoods for small p is hard. The standard deviation of fixed sampling is always lower than that of IBS, especially when $p \to 0$ (specifically when $p \ll \frac{1}{M}$). In other words, fixed sampling produces low-variance estimators exactly in the range in which its estimates are biased, as guaranteed by the Cramer-Ráo bound. However, in the large-M limit, fixed sampling does saturate the information inequality, so its master curve lies below IBS. In other words, if one is able to draw so many samples that bias is no issue, then fixed sampling provides a slightly better trade-off between variance and computational time. However, in Section C.2, we discuss an improvement to IBS which decreases its variance by a factor 2-20, in which case IBS is clearly superior. Finally, a quantity of interest for the researcher may not be the variance of the estimator per se, but a measure of the error such as the RMSE, for which IBS is also consistently superior (see Section A.6).

A.5 A Bayesian derivation of the IBS estimator

In the main text and Section A.2 we hinted at a Bayesian interpretation of the problem of estimating $\log p$. We show here that indeed we can see the IBS estimator as a Bayesian point estimate of $\log p$ with a specific choice of prior for p. For the rest of this section, we use q to denote the likelihood of a trial (instead of p); that is q is the parameter of the Bernoulli distribution and $\log q$ the quantity we are seeking to estimate. We changed notation to avoid confusion with expressions such as the prior probability of q, which is $p(q)$.

Let K be the number of samples until a 'hit', as per the IBS sampling policy. Following Bayes' rule, we can write the posterior over q given K as

$$\begin{aligned} p(q|K) &= \frac{\Pr(K|q)p(q)}{\Pr(K)} \\ &= \frac{(1-q)^{K-1} q \,\text{Beta}(q;\alpha,\beta)}{\int_0^1 (1-q)^{K-1} q \,\text{Beta}(q;\alpha,\beta) dq} \\ &= \frac{\Gamma(K+\alpha+\beta)}{\Gamma(\alpha+1)\Gamma(K+\beta-1)} (1-q)^{K+\beta-2} q^{\alpha}, \end{aligned} \tag{S5}$$

[1]These curves converge pointwise on $(0, 1]$ and uniformly on any interval $(\varepsilon, 1]$, but not uniformly on $(0, 1]$. The limits $M \to \infty$ and $p \to 0$ are not exchangeable.

where we used the fact that $\Pr(K|q)$ follows a geometric distribution, and we assumed a Beta(α, β) prior over q.

In particular, let us compute the posterior mean of $\log q$ under the Haldane prior, Beta$(0, 0)$ [5]. Thanks to the 'law of the unconscious statistician', we can compute the posterior mean of $p(\log q|K)$ directly from Eq S5,

$$\begin{aligned} \mathbb{E}_{p(\log q|K)}\left[\log q\right] &= (K-1)\int_0^1 (\log q)(1-q)^{K-2} dq \\ &= \int_0^1 (\log q)\text{Beta}(q; 1, K-1) dq \\ &= \psi(1) - \psi(K) \\ &= -\sum_{k=1}^{K-1} \frac{1}{k}, \end{aligned} \tag{S6}$$

where the first row follows from setting $\alpha = 0$ and $\beta = 0$; it can be shown that the third row is the expectation of $\log q$ for a Beta distribution, with $\psi(z)$ the *digamma function* [6]; and the last equality follows from the relationship between the digamma function and harmonic numbers, that is $\psi(n) = -\gamma + \sum_{k=1}^{n-1} \frac{1}{k}$, where γ is Euler-Mascheroni constant. We also used the notational convention that $\sum_{k=1}^{0} a_k = 0$ for any a_k. Note that the last row is equal to the IBS estimator, $\hat{\mathcal{L}}_{\text{IBS}}(K)$, as defined in Eq 14 in the main text.

Crucially, Eq S5 shows that we can recover the IBS estimator as the *posterior mean* of $\log q$ given K, under the Haldane prior for q. This interpretation allows us to also define naturally the *variance* of our estimate for a given K, as the variance of the posterior over $\log q$,

$$\text{Var}_{p(\log q|K)}\left[\log q\right] = \psi_1(1) - \psi_1(K), \tag{S7}$$

where $\psi_1(z)$ is the *trigamma function*, the derivative of the digamma function; the equality follows from a known expression for the variance of $\log q$ under a Beta distribution for q.

A.6 Estimator RMSE

In the main text and in previous comparisons we have discussed the *bias* and the *variance* of estimators of the log-likelihood, which are important statistical properties, but one might wonder how bias and variance combine to yield an error metric of practical relevance such as the *root mean squared error* (RMSE). Crucially, this analysis depends on the number of trials N (because bias and standard deviation scale differently with N) and on the distribution of values of the likelihood for different trials, p_i.

For illustrative purposes, we took as an example the psychometric model described in the Results section of the main text, and calculated the distribution of p_i for typical datasets and parameters settings. We then calculated the RMSE in estimating the total log-likelihood of a number of randomly generated datasets (sampled from the empirical distribution of p_i) with different number of trials; for different numbers of samples used by the IBS and fixed-sampling estimators.

Fig S4 shows that starting from even a handful of trials ($N = 10$), IBS is consistently better than fixed sampling at estimating the true value of the log-likelihood of a given parameter vector, and overwhelmingly so for a moderate number of trials ($N \geq 100$).

B Experimental details

In this section, we report details for the three numerical experiments described in the main text and supplementary results.

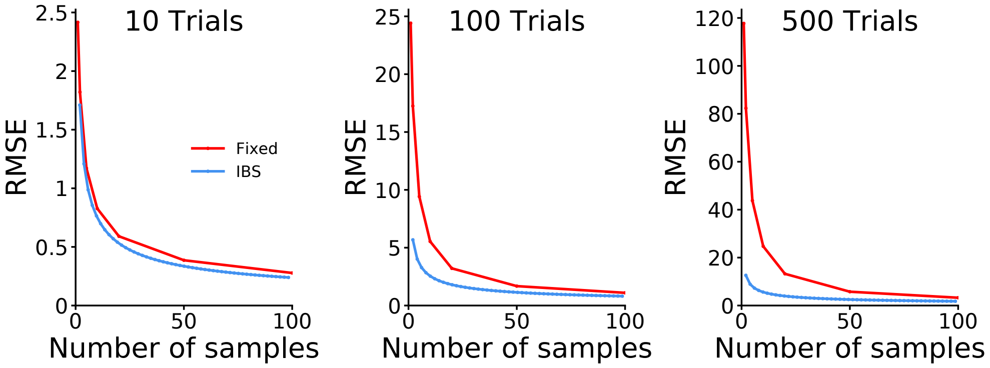

Figure S4. Log-likelihood RMSE. RMSE of the log-likelihood estimate as a function of number of samples, for the IBS and fixed-sampling estimators. Different panels display the RMSE curves for different number of trials.

B.1 Orientation discrimination

The parameters of the orientation discrimination model are the (inverse) slope, or sensory noise, represented as $\eta \equiv \log \sigma$, the bias μ, and the lapse rate γ. The logarithmic representation for σ is a natural choice for scale parameters [](and more in general, for positive parameters that can span several orders of magnitude).

We define the lower bound (LB), upper bound (UB), plausible lower bound (PLB), and plausible upper bound (PUB) of the parameters as per Table S1. The upper and lower bounds are hard constraints, whereas the plausible bounds provide information to the algorithm to where the global optimum is likely to be, and are used by BADS, for example, to draw a set of initial points to start building the surrogate Gaussian process, and to set priors over the Gaussian process hyperparameters [7]. Here we also use the plausible bounds to select ranges for the parameters used to generate simulated datasets, and to initialize the optimization, as described below.

Table S1. Parameters and bounds of the orientation discrimination model.

Parameter	Description	LB	UB	PLB	PUB
$\eta \equiv \log \sigma$	Slope	$\log 0.1$	$\log 10$	$\log 0.1$	$\log 5$
μ	Bias (°)	-2	2	-1	1
γ	Lapse	0.01	1	0.01	0.2

To generate synthetic data sets, we select 120 'true' parameter settings for the orientation discrimination task as follows. We set the baseline parameter $\boldsymbol{\theta}_0$ as $\eta = \log 2°$, $\mu = 0.1°$, and $\gamma = 0.1$. Then, for each parameter $\theta_j \in \{\eta, \mu, \gamma\}$, we linearly vary the value of θ_j in 40 increments from PLB_j to PUB_j as defined in Table S1 (e.g., from $-1°$ to $1°$ for μ), while keeping the other two parameters fixed to their baseline value. For each one of the 120 parameter settings $\boldsymbol{\theta}_{\text{true}}$ defined in this way, we randomly generated stimuli and responses for 100 datasets from the generative model, resulting in 12000 distinct data sets for which we know the true generating parameters.

We evaluated the log-likelihood with the following methods: fixed sampling with M samples, with $M \in \{1, 2, 3, 5, 10, 15, 20, 35, 50, 100\}$; IBS with R repeats, with $R \in \{1, 2, 3, 5, 10, 15, 20, 35, 50\}$; and exact. To avoid wasting computations on particularly 'bad' parameter settings, for IBS we used the 'early stopping threshold' technique described in Section C.1, setting a lower bound on the log-likelihood of IBS equal to the log-likelihood of a chance model, that is $\mathcal{L}_{\text{lower}} = -N \log 2$. While this might seemingly provide an advantage to IBS with respect to Fixed sampling, note that it is simply a way to ameliorate a weakness of IBS (spending too much time on 'bad' parameters vectors, which are largely inconsequential for optimization), which Fixed does not suffer from. Even

so, the stopping threshold was rarely reached (2% of evaluations).

For each data set and method, we optimized the log-likelihood by running BADS 8 times with different starting points. We selected starting points as the points that lie on one-third or two-third of the distance between the plausible upper and lower bound for each parameter, that is all combinations of $\eta \in \{-0.998, 0.305\}$, $\mu \in \{-0.333°, 0.333°\}$, $\gamma \in \{0.073, 0.137\}$. Each of these optimization runs returns a candidate for $\widehat{\boldsymbol{\theta}}_{\text{MLE}}$. For methods that return a noisy estimate of the log-likelihood, we then re-evaluate $\hat{\mathcal{L}}(\boldsymbol{\theta})$ for each of these 8 candidates with higher precision (for fixed sampling, we use $10M$ samples; for IBS, we use $10R$ repeats). Finally, we select the candidate with highest (estimated) log-likelihood.

When estimating parameters using IBS or fixed sampling, we enabled the 'uncertainty handling' option in BADS, informing it to incorporate measurement noise into its model of the objective function. Note that during the optimization, the algorithm iteratively infers a single common value for the observation noise σ_{obs} associated with the function values in a neighborhood of the current point [7]. A future extension of BADS may allow the user to explicitly provide the noise associated with each data point, which is easily computed for the IBS estimates (Eq 16 in the main text), affording the construction of a better surrogate model of the log-likelihood.

Alternative fixed sampling estimator

In the main text, we considered the fixed-sampling estimator defined by Eq 11. We performed an additional analysis to empirically validate that our results do not depend on the specific choice of estimator for fixed sampling (as expected given the theoretical arguments in the Methods section "Why fixed sampling fails" of the main text).

An alternative way of avoiding the divergence of fixed sampling is to correct samples that happen to be all zeros, for example with

$$\hat{\mathcal{L}}_{\text{fixed-bound}}(\boldsymbol{x}) = \log\left(\frac{\max\{m(\boldsymbol{x}), m_{\min}\}}{M}\right), \tag{S8}$$

for some $0 < m_{\min} < 1$, which sets a lower bound for the log-likelihood equal to $\log(m_{\min}/M)$. We then performed our analyses of the orientation discrimination task using the $\hat{\mathcal{L}}_{\text{fixed-bound}}$ estimator with $m_{\min} = \frac{1}{2}$. As shown in Fig S5, the results are remarkably similar to what we found using the fixed-sampling estimator $\hat{\mathcal{L}}_{\text{fixed}}$ defined by Eq 11 in the main text. Finally, we also tried $\hat{\mathcal{L}}_{\text{fixed-bound}}$ with a small value $m_{\min} = 10^{-3}$, which yielded even worse results (data not shown).

Complete parameter recovery results

For completeness, we report in Fig S6 the parameter recovery results for fixed sampling, inverse binomial sampling and 'exact' analytical methods for the orientation discrimination task, for all tested number of samples M and IBS repeats R. All estimates were obtained via maximum-likelihood estimation using the Bayesian Adaptive Direct Search [7], as described previously in this section.

B.2 Change localization

First, we derive the trial likelihood of the change localization model. Assuming that the change happens at location $c \in \{1, \ldots, 6\}$, by symmetry we can write

$$\Pr(\text{respond } i | c \text{ changed}) = \begin{cases} P_{\text{correct}}\left(\Delta_s^{(c)}; \boldsymbol{\theta}\right) & \text{if } i = c \\ \frac{1}{5}\left(1 - P_{\text{correct}}\left(\Delta_s^{(c)}; \boldsymbol{\theta}\right)\right) & \text{otherwise} \end{cases} \tag{S9}$$

where $\Delta_s^{(c)} = \left| d_{\text{circ}}(s_c^{(1)}, s_c^{(2)}) \right|$ is the absolute circular distance between the true orientations of patch c in the first and second display. We can derive an expression for $P_{\text{correct}}\left(\Delta_s^{(c)}; \boldsymbol{\theta}\right)$ by marginalizing over the circular distance

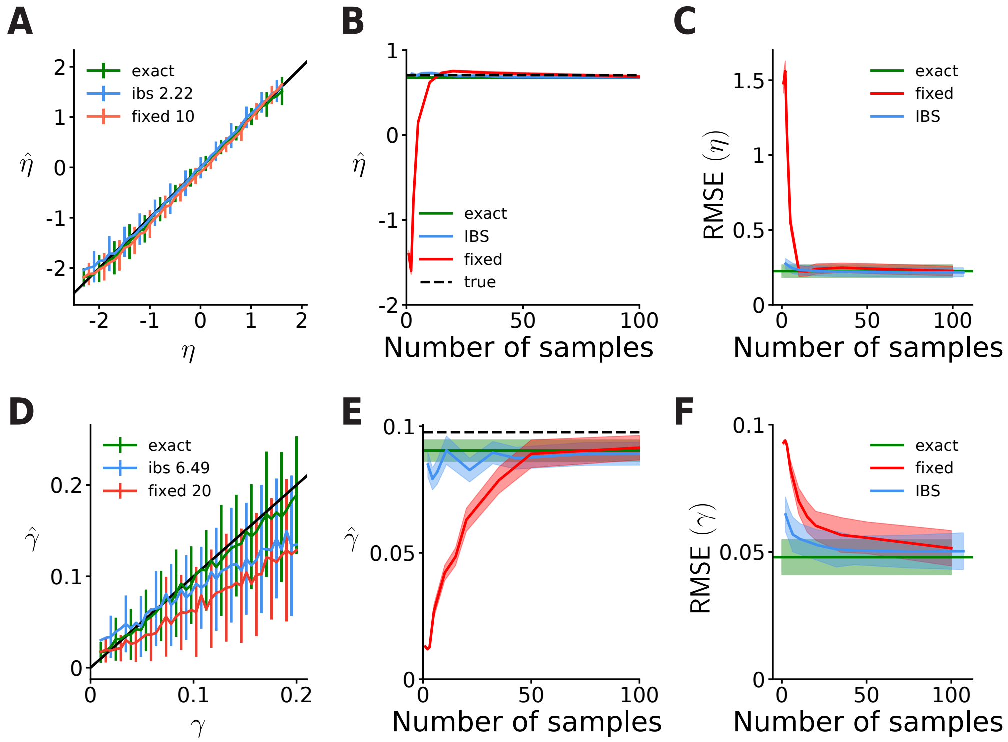

Figure S5. Parameter recovery for the orientation discrimination model with alternative fixed sampling estimator. Same as Fig 5 in the main text, but for the alternative fixed-sampling estimator defined by Eq S8. The results are qualitatively identical.

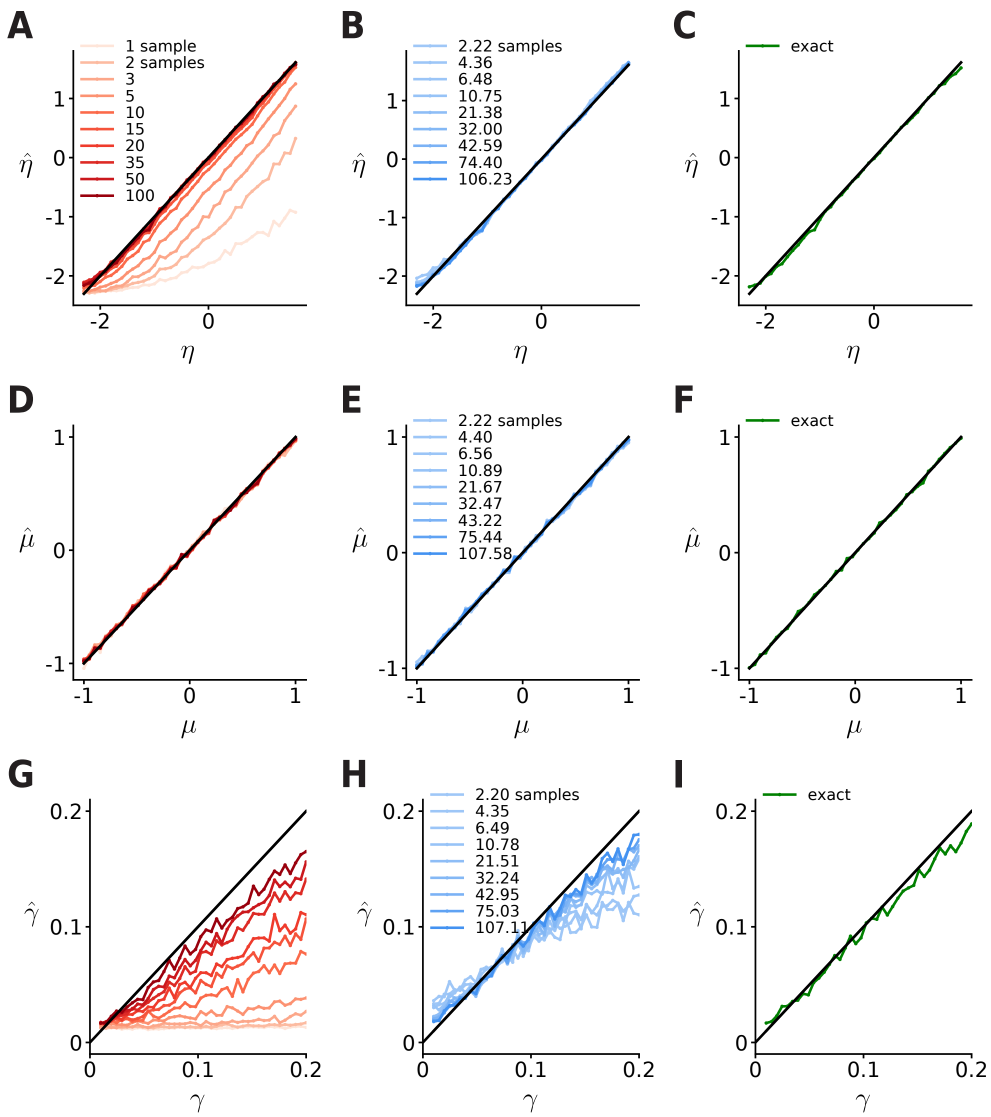

Figure S6. Full parameter recovery results for the orientation discrimination model. A. Mean estimates recovered by fixed sampling with different number of samples. Error bars are omitted to avoid visual clutter. **B.** Mean estimates recovered by IBS with different numbers of repeats. The legend reports the average number of samples per trial that IBS uses to obtain these estimates. **C.** Mean estimate recovered using the 'exact' log-likelihood function (see Eq 20 in the main text). **D-F** Same, for the bias parameter μ. **G-I** Same, for the lapse rate γ. Overall, fixed sampling produces highly biased estimates of η and γ, while IBS is much more accurate. The bias parameter μ can be accurately estimated by either method regardless of the number of samples or repeats.

between the respective measurements,

$$P_{\text{correct}}\left(\Delta_s^{(c)};\boldsymbol{\theta}\right)=\frac{\gamma}{6}+(1-\gamma)\int_0^{2\pi}\Pr\left(\Delta_x^{(c)}|\Delta_s^{(c)}\right)\Pr\left(\forall i\neq c:\Delta_x^{(i)}\leq\Delta_x^{(c)}|\Delta_s^{(i)}=0\right)\mathrm{d}\Delta_x^{(c)},\qquad\text{(S10)}$$

where we have defined $\Delta_x^{(i)}=\left|d_{\text{circ}}(x_i^{(1)},x_i^{(2)})\right|$ and we suppressed the dependence on κ to simplify the notation. The first term in this equation is the probability density function (pdf) of the circular distance between two von Mises random variables whose centers are $\Delta_s^{(j)}$ apart. The second term simplifies, since $\Delta_x^{(i)}$ for all $i\neq j$ are all independent and identically distributed. Therefore, we can rewrite this equation as

$$P_{\text{correct}}\left(\Delta_s^{(c)};\boldsymbol{\theta}\right)=\frac{\gamma}{6}+(1-\gamma)\int_0^{2\pi}\Pr\left(\Delta_x^{(c)}|\Delta_s^{(c)}\right)\Pr\left(\Delta\leq\Delta_x^{(c)}\right)^5\mathrm{d}\Delta_x^{(c)},\qquad\text{(S11)}$$

where Δ is an auxiliary variable generated by taking the absolute circular difference between two von Mises random variables that are centered at 0 with concentration parameter κ. The second term of the integrand, therefore, is the fifth power of the cumulative distribution function (cdf) of Δ. We can compute the distribution of the circular distance between two von Mises random variables analytically, but the cdf is non-analytic. Moreover, the integral in Eq S11 is analytically intractable as well. We can, however, evaluate it numerically via trapezoidal integration (see Fig 6 in the main text).

We now describe the settings used for maximum-likelihood estimation. The model parameters are the sensory noise, represented as $\eta\equiv\log\sigma$ (with $\sigma=\frac{1}{\sqrt{\kappa}}$), and the lapse rate γ, with bounds defined in Table S2. We use the same procedure and settings for BADS as for the orientation discrimination task (see Section B.1). For IBS, we use an early-stopping threshold of $\mathcal{L}_{\text{lower}}=-N\log 6$, and we use repeats $R\in\{1,2,3,5,10,15,20\}$ (since due to the larger response space IBS uses more samples per run). We run BADS 4 times, with starting values of $\eta\in\{-1.535,-0.767\}$ and $\gamma\in\{0.173,0.337\}$. For data generation, we select 40 parameter vectors with $\eta=\log 0.3$ and γ linearly spaced from 0.01 to 0.5 and 40 data sets with $\gamma=0.03$ and η between $\log 0.1$ and $\log 1$. Again, we generate 100 data sets for each such parameter combination.

Table S2. Parameters and bounds of the change localization model.

Parameter	Description	LB	UB	PLB	PUB
$\eta\equiv\log\sigma$	Sensory noise	$\log 0.05$	$\log 2$	$\log 0.1$	$\log 1$
γ	Lapse	0.01	1	0.01	0.5

Complete parameter recovery results

We report in Fig S7 the parameter recovery results for fixed sampling, inverse binomial sampling and 'exact' methods for the change localization task, for all tested number of samples M and IBS repeats R. For this task, the exact method relies on numerical integration.

B.3 Four-in-a-row game

The four-in-a-row game model parameters are the value noise $\eta\equiv\log\sigma$, the pruning threshold ξ, and the feature dropping rate δ, with bounds defined in Table S3. We use the same procedure and settings for BADS as for the orientation discrimination task (see Section B.1), unless noted otherwise. For IBS, we use an early-stopping threshold of $\mathcal{L}_{\text{lower}}=-N\log 20$, and due to computational cost we use only $R\in\{1,2,3\}$. For fixed sampling we consider $M\in\{1,2,3,5,10,15,20,35,50,100\}$. We have no expression for the likelihood of the four-in-a-row game model, not even in numerical form, so there is no 'exact' method.

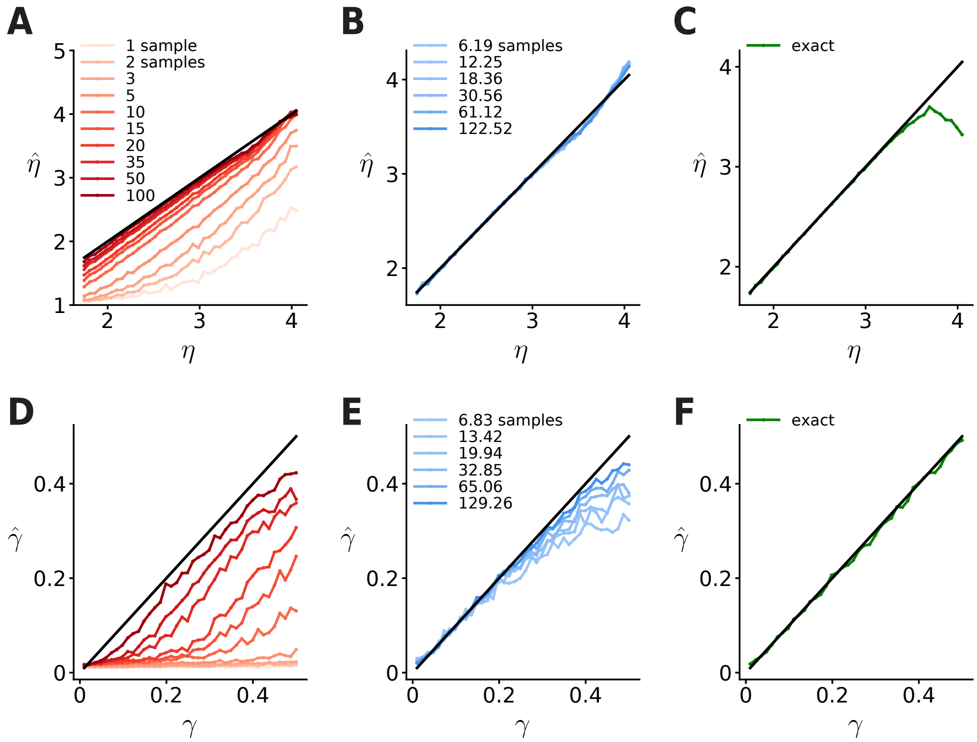

Figure S7. Full parameter recovery results for the change localization model. Same as Fig S6, for the change localization model. Fixed sampling is substantially biased for both the measurement noise η and the lapse rate γ, whereas IBS is accurate for η and biased for γ, but still much less biased than fixed sampling.

Table S3. Parameters and bounds of the four-in-a-row game model.

Parameter	Description	LB	UB	PLB	PUB
$\eta \equiv \log \sigma$	Value noise	$\log 0.01$	$\log 5$	$\log 0.2$	$\log 3$
ξ	Pruning threshold	0.01	10	1	10
δ	Feature dropping rate	0	1	0	0.5

We run BADS 8 times, with starting values of $\eta \in \{-0.707, 0.196\}$, $\xi \in \{4, 7\}$ and $\delta \in \{0.167, 0.333\}$. For data generation, we set as baseline parameter vector $\eta = \log 1$, $\xi = 5$ and $\delta = 0.2$ and for each parameter we select 40 parameter vectors linearly spaced in the plausible range for that parameter (as per Table S3), while keeping the other two parameters at their baseline value. Again, we generate 100 data sets for each such parameter combination.

We fixed the other parameters of the model to typical values found in the previous study [8], namely $w_{\text{center}} = 0.60913$, $w_{\text{connected 2-in-a-row}} = 0.90444$, $w_{\text{unconnected 2-in-a-row}} = 0.45076$, $w_{\text{3-in-a-row}} = 3.4272$, $w_{\text{3-in-a-row}} = 6.1728$, $C_{\text{act}} = 0.92498$, $\gamma_{\text{tree}} = 0.02$, $\lambda = 0.05$. The w_i are the weights of features f_i in the value function, briefly f_{center} values pieces near the center of the board, the other features count the number of times certain patterns occur on the board (see [8] for the specific patterns). C_{act} is a parameter which scales the value of features belonging to the active or passive player. The parameter γ_{tree} is inversely proportional to the size of the tree built by the algorithm, and λ is the lapse rate, that is the probability of a uniformly random move among the available squares (note that for the other models we denoted lapse rate as γ; here we use the variable naming from [8]). See [8] for more details about the model and its parameters.

Complete parameter recovery results

We report in Fig S8 the parameter recovery results for fixed sampling and inverse binomial sampling for the 4-in-a-row task, for all tested number of samples M and IBS repeats R. For this task, there is no 'exact' method to evaluate the log-likelihood.

C Improvements of IBS and further applications

C.1 Early stopping threshold

One downside of inverse binomial sampling is that the computational time it uses to estimate the log-likelihood is of the order of $\frac{1}{p}$, which is equal to $\exp(-\log p) = \exp(-\mathcal{L})$. In other words, IBS spends exponentially more time on estimating log-likelihoods of poorly-fitting models or bad parameters. This implies that an optimization algorithm that uses IBS allocates more computational resources to estimating the objective function $\mathcal{L}(\boldsymbol{\theta})$ for parameter vectors $\boldsymbol{\theta}$ where the objective is low. However, the value of the objective at such poor parameter vectors are unlikely to affect its estimate of the location or value of the maximum, so the optimizer (BADS in our case) is wasting time. It may be possible to develop optimization algorithms that take into account the exponentially large cost of probing points where the objective function is low, but we can circumvent the problem by amending IBS with a criterion that stops sampling when it realizes that $\hat{\mathcal{L}}(\boldsymbol{\theta})$ will be low.

In the Methods section of the main text, we introduced a basic implementation of IBS for estimating the log-likelihood of multiple trials, by sequentially computing the log-likelihood of each trial. However, another way to implement multi-trial IBS (a 'parallel' implementation) is to draw one sample from the simulator model for each trial, then set $K_i = 1$ for each trial where the sample matches the participant's response. For all other trials, draw a second sample from the model, and if that matches the response, set $K_i = 2$. Finally, repeat this process until no more trials remain. We illustrate this sampling scheme graphically in Fig S9.

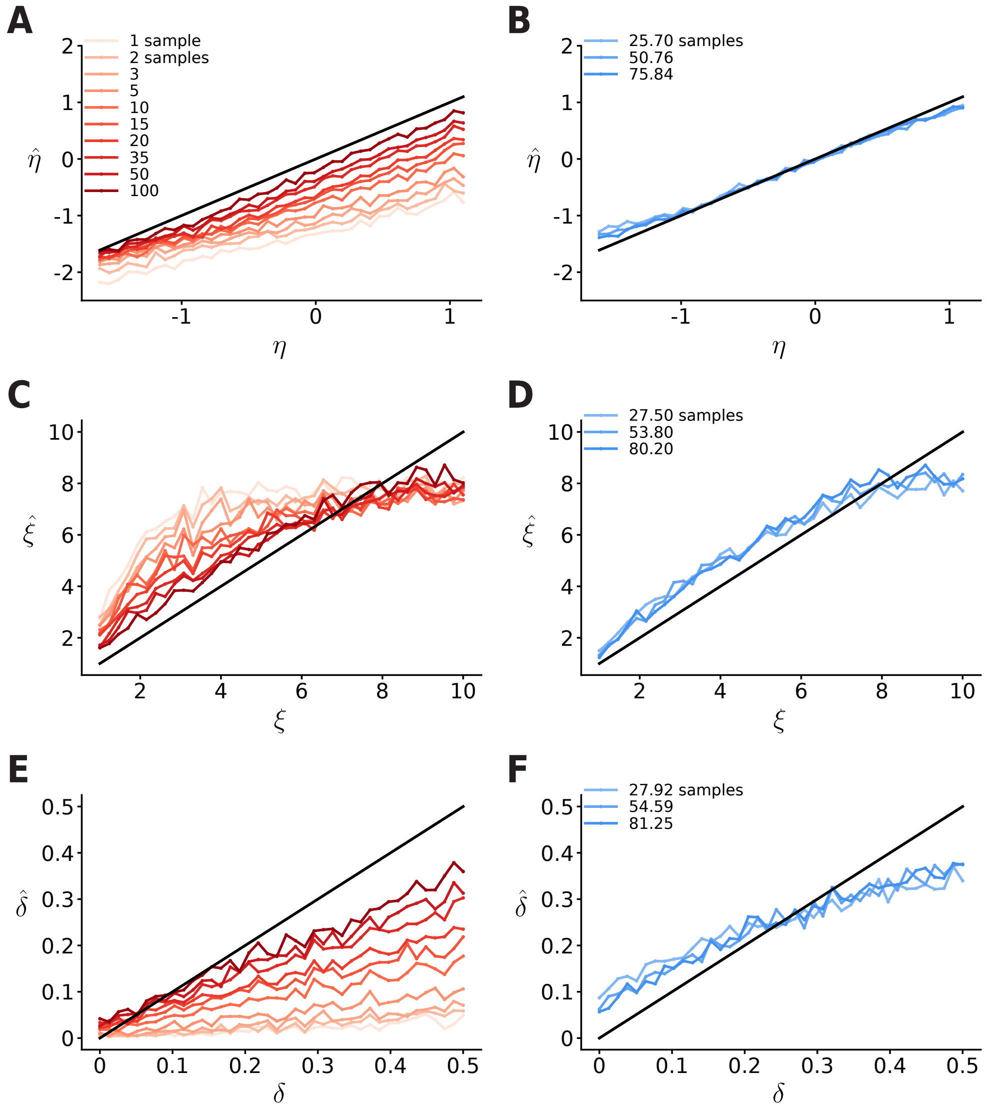

Figure S8. Full parameter recovery results for the four-in-a-row model. Same as Fig S6, for the four-in-a-row task. For this model, we do not have an exact log-likelihood formula or numerical approximation, so we only show fixed sampling and IBS. Overall, fixed sampling has substantial biases in its estimation of η and δ and a smaller bias in estimating ξ. IBS has almost no bias for η and only a small bias for ξ and δ.

After each iteration, we then compute

$$\hat{\mathcal{L}}_K = -\sum_{i \in \mathcal{I}_{\text{match}}} \sum_{k=1}^{K_i-1} \frac{1}{k} - N_{\text{remaining}} \sum_{k=1}^{K-1} \frac{1}{k} \tag{S12}$$

where K is the iteration number, $\mathcal{I}_{\text{match}}$ is the set of trials where we found a matching sample and $N_{\text{remaining}}$ is the number of remaining trials. This value $\hat{\mathcal{L}}_K$ is decreasing and by construction converges to $\sum_{i=1}^{N} \hat{\mathcal{L}}_{i,\text{IBS}}$ as $K \to \infty$. Therefore, whenever $\hat{\mathcal{L}}_K$ falls below a lower bound $\mathcal{L}_{\text{lower}}$, we are guaranteed that $\hat{\mathcal{L}}_{\text{IBS}}$ will be below that bound too. When it does, we stop sampling and return $\mathcal{L}_{\text{lower}}$ as estimate of $\mathcal{L}(\boldsymbol{\theta})$. This does introduce bias into the estimate, but since we bound the *total* log-likelihood, the bias will be exponentially small in N as long as the true value $\mathcal{L}(\boldsymbol{\theta})$ is adequately larger than $\mathcal{L}_{\text{lower}}$.

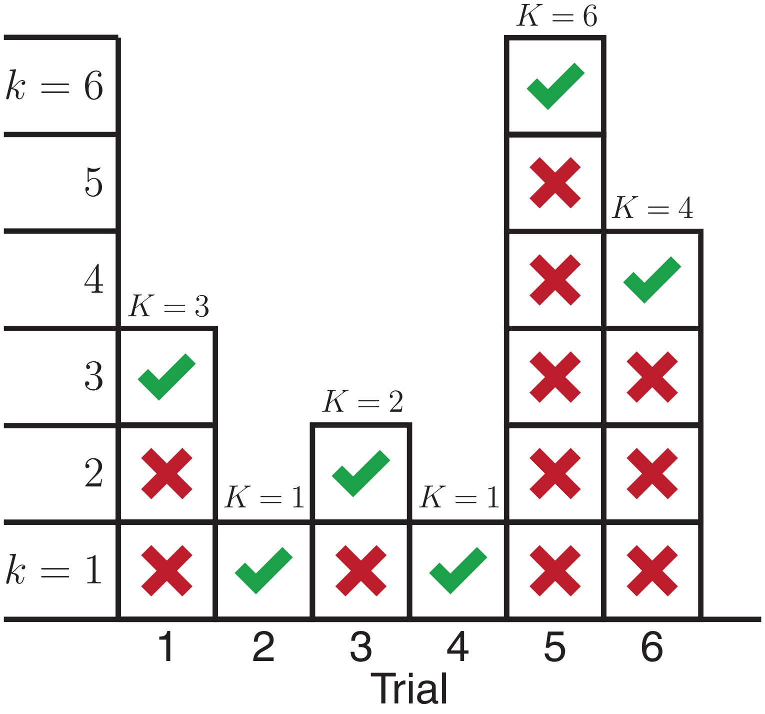

Figure S9. IBS implementation with multiple trials. Graphical illustration of the two methods to implement IBS with multiple trials, in this case $N = 6$. In this figure, each column represents a trial, each box above the trial number represents a successive sample from the model from that trial, with red crosses for samples that do not match the participant's response ('misses') and green checkmarks for ones that do ('hits'). Above each column, we indicate K, the number of samples until a hit. For trials 2 and 4, $K = 1$ so $\hat{\mathcal{L}}_{\text{IBS}} = 0$. The most obvious implementation of multi-trial IBS is 'columns-first', to sample model responses for each trial until a hit, and only then move to the next trial. However, a more convenient sampling method is 'rows-first', and sample one response for each trial with $k = 1$, then one response for each trial with $k = 2$, excluding trials 2 and 4 since the first sample was a hit, and continue increasing k until all trials reach a hit. This method allows for early stopping and a parallel processing.

In practice, we recommend using as lower bound the log-probability of the data under a 'chance' model, which assigns uniform probability to each possible response on each trial, and should be a poor model of the data. In the orientation discrimination and change localization examples from the Results section of the main text, the

log-likelihood of a chance model is $-N \log 2$ and $-N \log 6$, respectively. For the four-in-a-row game, the log-likelihood of chance depends on the number of pieces on each board position; we chose an average value such that $\mathcal{L}_{\text{lower}} = -N \log 20$. This new sampling scheme has an additional advantage: since on each iteration we independently sample from the generative model on multiple trials, we can potentially run these computations in parallel.

C.2 Reducing variance by trial-dependent repeated sampling

As we saw in the Methods section of the main text, a simple method to improve the estimate of IBS is to run the estimator multiple times and average the results. Repeated sampling will preserve the zero bias but reduce variance inversely proportional to the number of repeats R.

We can further improve the estimator by varying the number of repeats R_i between trials, for $1 \leq i \leq N$, and define

$$\hat{\mathcal{L}}_{\text{IBS-}\boldsymbol{R}} = \sum_{i=1}^{N} \frac{1}{R_i} \sum_{r=1}^{R_i} \hat{\mathcal{L}}_i^{(r)}, \tag{S13}$$

where $\boldsymbol{R}$ is a vector of positive integers with elements R_i, and $\hat{\mathcal{L}}_i^{(r)}$ denotes the outcome of the r-th run of IBS on trial i. This estimator is unbiased regardless of the choice of $\boldsymbol{R}$ (as long as $R_i > 0$ for all trials), and we can analytically compute both its variance and expected number of samples (see Eq S14).

We can then ask what is the best allocation of repeats R_i that minimizes the variance of the estimator in Eq S13 such that the expected total number of samples does not exceed a fixed budget S. This defines the following constrained optimization problem,

$$\boldsymbol{R}^* = \arg \min_{R_i, R_2 \ldots, R_N} \left\{ \frac{1}{N} \sum_{i=1}^{N} \frac{\text{Li}_2(1-p_i)}{R_i} \middle| \sum_{i=1}^{N} \frac{R_i}{p_i} \leq S \right\} \tag{S14}$$

where we used Eq 15 for the variance of the IBS estimator.

Assuming that the R_i take continuous values, we can solve the optimization problem in Eq S14 exactly using a Lagrange multiplier, and find the following closed-form expression for the optimal number of repeats per trial,

$$R_i^* = S \left(\sum_{j=1}^{N} \sqrt{\frac{\text{Li}_2(1-p_j)}{p_j}} \right)^{-1} \sqrt{p_i \text{Li}_2(1-p_i)}. \tag{S15}$$

According to Eq S15, the optimal choice of repeats entails dividing the budget S across trials, where trial i is allocated repeats proportional to $\sqrt{p_i \text{Li}_2(1-p_i)}$. We plot this function in Fig S10 and see that, to minimize variance, we should allocate resources primarily to trials where p_i is close to $\frac{1}{2}$ and avoid trials where $p_i \approx 1$ (since the variance of IBS is already small for those trials) or where $p_i \approx 0$ (since those utilize a larger share of the budget).

We can also calculate exactly the fractional increase in precision (inverse variance) when using the optimal choice of repeats vector $\boldsymbol{R}^*$, compared to a constant R which divides the budget equally across trials,

$$\frac{\text{Var}\left[\hat{\mathcal{L}}_{\text{IBS-}R}\right]}{\text{Var}\left[\hat{\mathcal{L}}_{\text{IBS-}\boldsymbol{R}^*}\right]} = \left(\sum_{i=1}^{N} \sqrt{\frac{\text{Li}_2(1-p_i)}{p_i}} \right)^2 \times \left(\sum_{i=1}^{N} \text{Li}_2(1-p_i) \right)^{-1} \times \left(\sum_{i=1}^{N} \frac{1}{p_i} \right)^{-1}. \tag{S16}$$

This equation implies that the gain in precision from this method depends on the distribution of p_i across trials. If $p_i \sim$ Uniform[0,1] and $N = 500$, the median precision gain is 1.584 and the inter-quartile range (IQR) is 1.375 to 2.090. Note that the gain is always greater than 1, unless p_i is constant across trials.

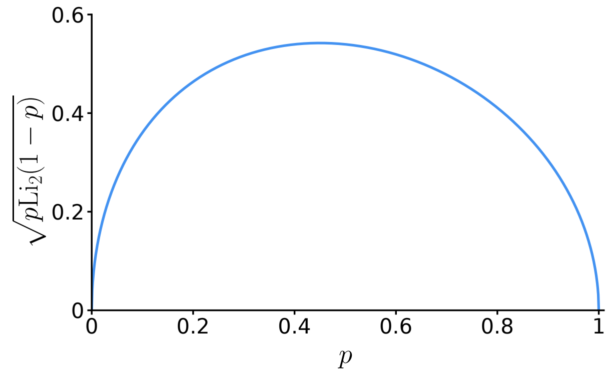

Figure S10. Optimal number of repeats. Graph of $\sqrt{p\mathrm{Li}_2(1-p)}$, which is proportional to the optimal number of repeats for a trial with likelihood p (see equation S15). We observe that the optimal allocation of computational resources entails repeated sampling for trials with $p \approx \frac{1}{2}$ and to avoid $p \approx 0$ or $p \approx 1$.

Practical implementation of trial-dependent repeated sampling

In practice, Eq S15 cannot be applied directly, as we have treated R_i as continuous variables, but the number of times to repeat IBS on a given trial has to be an integer. Additionally, the method is only unbiased if R_i is at least 1 for each trial i. Therefore, we can convert R_i^* to integers by rounding up to the nearest integer. This method will make our solution approximate, and reduce the gain in precision, but it is still better than uniform repeats for uniformly distributed p_i (median: 1.567, IQR: 1.374-2.002).

The derivation above has another, more fundamental problem. Computing R_i^* requires knowledge of p_i on each trial, which we do not have. While we could try and learn the allocation of $\boldsymbol{R}^*$ as a function of $\boldsymbol{\theta}$ in some adaptive way, in practice we recommend the following simple scheme:

1. Choose a default parameter vector $\boldsymbol{\theta}_0$, and run IBS with a large number of repeats (e.g, $R = 100$) to estimate the (log)-likelihood of the model on each trial.
2. Compute the optimal repeats R_i^* given the estimated likelihoods $\hat{p}_i$ and a total budget of expected samples S per likelihood evaluation, and round up.
3. Run IBS with those fixed repeats per trial on each iteration of the optimization algorithm.

This approach implicitly assumes that the log-likelihood will be correlated across trials between the generative model with parameter vector $\boldsymbol{\theta}_0$ and any other vector $\boldsymbol{\theta}$ probed by the optimization algorithm. This is usually the case, since low-probability trials are often those where something unexpected occurred (e.g., the participant of a behavioral experiment lapsed or otherwise made an error). In our experience, this scheme considerably reduces the variance of IBS for a given computational time budget.

C.3 Bayesian inference with IBS

While the main text focused on maximum-likelihood estimation, the unbiased log-likelihood estimates provided by IBS can also be used to perform Bayesian inference of posterior distributions over model parameters. We describe here a few possible approaches to approximate Bayesian inference with IBS.

Markov Chain Monte Carlo

Markov Chain Monte Carlo (MCMC; see e.g. [9]) is a powerful class of algorithms that allows one to sequentially sample from a target probability density which is known up to a normalization constant (e.g., the joint distribution).

A popular form of MCMC is known as Metropolis-Hastings (MH [10]), which explores the target distribution by drawing a sample from a 'proposal distribution' centered on the last sample (e.g., a multi-variate Gaussian). MH 'accepts' or 'rejects' the new sample with an acceptance probability that depends on the value of the target density at the proposed and at the last point. In case of acceptance, the new point is added the sequence of samples; otherwise, the last sample is repeated in the sequence. Under some conditions, the MH algorithm produces a (correlated) sequence of samples that are equivalent to draws from the target density. Crucially, and somewhat surprisingly, the MH algorithm is still valid (that is, produces a valid sequence) if one performs the comparison with a noisy but unbiased estimate of the target density as opposed to using the exact density [11].

One problem here is that IBS provides an unbiased estimate of the log-likelihood (and thus of the log target density); not of the likelihood. However, since the IBS estimates of the log-likelihood are nearly-exactly normally distributed (see Fig 3 in the main text), the distribution of the likelihood is log-normal. Thus, we can apply what is known as a 'convexity correction' and compute a (nearly) unbiased estimate of the likelihood $\hat{\ell}(\boldsymbol{\theta})$ by calculating the expected value of a log-normal variable, that is

$$\hat{\ell}(\boldsymbol{\theta}) = \exp\left(\hat{\mathcal{L}}(\boldsymbol{\theta}) + \frac{1}{2}\mathrm{Var}\left[\hat{\mathcal{L}}(\boldsymbol{\theta})\right]\right). \tag{S17}$$

Eq S17 can be easily evaluated with IBS, using the expression for the variance of the IBS estimator (Eq 16 in the main text).

Variational inference

An alternative class of approximate inference methods is based on *variational inference* (VI [12]). The goal of VI is to approximate the intractable posterior distribution with a simpler distribution $q(\boldsymbol{\theta})$ belonging to a chosen parametric family. A common choice is a multivariate normal with diagonal covariance (known as *mean field approximation*); but other choices are possible too. VI selects the 'best' approximation $q(\cdot)$ that minimizes the Kullback-Leibler divergence with the true posterior, or equivalently maximizes the following variational objective,

$$\mathcal{E}\left[q\right] = \mathbb{E}_{\boldsymbol{\theta}\sim q(\cdot)}\left[\mathcal{L}(\boldsymbol{\theta})\right] + \mathcal{H}\left[q\right], \tag{S18}$$

where $\mathcal{H}\left[q\right]$ is the entropy of $q(\cdot)$, which we assume can be computed analytically or numerically. Crucially, we can obtain an unbiased estimate of the first term in Eq S18 (the expected log joint) with IBS, as we have seen in the Discussion in the main text. The optimization of the variational objective can then be performed directly with derivative-free optimization methods (such as BADS), or via a technique that produces unbiased estimates of the gradient combined with variance-reduction tricks, called *black-box variational inference* [13].

Gaussian process surrogate methods

One issue with the approximate inference methods described above is that they require a large (possibly, *very* large) number of likelihood evaluations to converge. Thus, these approaches are unfeasible if the generative model is somewhat computationally expensive, as it is often the case. An alternative family of methods designed to deal with expensive likelihoods builds a Gaussian process approximation (a surrogate) of the log joint distribution, and uses it to actively acquire further points in a smart way, similarly to the approach of Bayesian optimization [14–16]. However, unlike Bayesian optimization, the goal here is not to *optimize* the target function, but instead to build an accurate approximation of the posterior distribution, with as few likelihood evaluations as possible.

IBS is particularly suited to be used in combination with Gaussian process surrogate methods as it provides both an unbiased estimate of the log-likelihood, and a calibrated estimate of the uncertainty in each measurement, which can be used to inform the Gaussian process observation model. The development of Gaussian process surrogate methods is an active and very promising area of research. A recent example is Variational Bayesian Monte Carlo (VBMC [15, 17]), a technique that naturally combines Gaussian process surrogate modeling with variational inference thanks to Bayesian quadrature [18]. Conveniently, VBMC returns both an approximate posterior distribution and an estimate of the model evidence, which can be used for model comparison. Recent work showed that

VBMC, combined with IBS and modified to deal with noisy log-likelihood evaluations, performs very well on a variety of models from computational and cognitive neuroscience [19].

Supplemental References

1. de Groot MH. Unbiased sequential estimation for binomial populations. The Annals of Mathematical Statistics. 1959;30(1):80–101.
2. Lehmann EL, Casella G. Theory of point estimation. Springer Science & Business Media; 2006.
3. Kolmogorov AN. Unbiased esimates. Izvestiya RAN Seriya Matematicheskaya. 1950;14(4):303–326.
4. Dawson R. Unbiased tests, unbiased estimators, and randomized similar regions. Harvard University; 1953.
5. Haldane JBS. A note on inverse probability. In: Mathematical Proceedings of the Cambridge Philosophical Society. vol. 28. Cambridge University Press; 1932. p. 55–61.
6. Abramowitz M, Stegun IA. Handbook of mathematical functions with formulas, graphs, and mathematical tables. vol. 55. US Government Printing Office; 1948.
7. Acerbi L, Ma WJ. Practical Bayesian Optimization for Model Fitting with Bayesian Adaptive Direct Search. In: Advances in Neural Information Processing Systems; 2017. p. 1836–1846.
8. van Opheusden B, Bnaya Z, Galbiati G, Ma WJ. Do people think like computers? In: International Conference on Computers and Games. Springer; 2016. p. 212–224.
9. Brooks S, Gelman A, Jones G, Meng XL. Handbook of Markov Chain Monte Carlo. CRC press; 2011.
10. Hastings WK. Monte Carlo sampling methods using Markov chains and their applications. Biometrika. 1970;57(1):97–109.
11. Andrieu C, Roberts GO, et al. The pseudo-marginal approach for efficient Monte Carlo computations. The Annals of Statistics. 2009;37(2):697–725.
12. Jordan MI, Ghahramani Z, Jaakkola TS, Saul LK. An introduction to variational methods for graphical models. Machine Learning. 1999;37(2):183–233.
13. Ranganath R, Gerrish S, Blei D. Black box variational inference. In: Artificial Intelligence and Statistics; 2014. p. 814–822.
14. Kandasamy K, Schneider J, Póczos B. Bayesian active learning for posterior estimation. In: Twenty-Fourth International Joint Conference on Artificial Intelligence; 2015.
15. Acerbi L. Variational Bayesian Monte Carlo. In: Advances in Neural Information Processing Systems; 2018. p. 8213–8223.
16. Järvenpää M, Gutmann M, Vehtari A, Marttinen P. Parallel Gaussian process surrogate method to accelerate likelihood-free inference. arXiv preprint arXiv:190501252. 2019.
17. Acerbi L. An Exploration of Acquisition and Mean Functions in Variational Bayesian Monte Carlo. In: Symposium on Advances in Approximate Bayesian Inference; 2019. p. 1–10.
18. O'Hagan A. Bayes–Hermite quadrature. Journal of Statistical Planning and Inference. 1991;29(3):245–260.
19. Acerbi L. Variational Bayesian Monte Carlo with Noisy Likelihoods. arXiv preprint arXiv:200608655. 2020.
