# Supplementary material for: Unbiased and efficient log-likelihood estimation with inverse binomial sampling
Source: PLoS Comput Biol. 2020 Dec 23;16(12):e1008483. doi: 10.1371/journal.pcbi.1008483 (PMC7758077; doi:10.1371/journal.pcbi.1008483)
Supplement: Attachment — Submitted filename: ResponseToReviewers.pdf [file pcbi.1008483.s002.pdf]

**Unbiased and Efficient Log-Likelihood Estimation with Inverse Binomial Sampling**

**Response to reviewers**

We thank the reviewers for the useful feedback on our manuscript. In response to the reviewers' comments and suggestions, we performed the following changes, sanity checks, and additions to the manuscript, which we feel address the concerns raised:

1. Following the reviewers' comments and suggestions, we rewrote and added several paragraphs to improve clarity and provide better explanations for our methods and results. We report major changes and additions in the rest of this document, and we attach to the revision a "diffed" manuscript with highlighted changes (*please find the download link at the end of the revised submission PDF*).
2. We added the derivation of Equation 15 (variance of the IBS estimator) to the Appendix.
3. We remade several figures for the revised manuscript, and made new figures to answer reviewers' questions (included in this document).
4. Upon request of Reviewer #2, we additionally tested IBS for estimating the parameters of a heavy-tailed distribution (included in this document).
5. Upon request of Reviewer #3, we ran an additional study with a different Fixed estimator.
6. To answer concerns of both Reviewers #1 and #3 regarding the early-stopping threshold, we reran the analysis in the main text with the Psychometric model and counted how often the threshold was actually achieved (see discussion later).
7. Finally, we reformatted the manuscript under the requirements of *PLoS Computational Biology*, as instructed by the PLoS editorial team (e.g., we separated the Appendix from the main text, we removed section numbers, changed the referencing system, etc.).

We believe that with these additions and further validations, and by making our data and code fully available, our manuscript brings a valuable contribution to the readers of *PLoS Computational Biology*, by providing a technique that affords efficient and unbiased log-likelihood estimation, opening the entire toolbox of likelihood-based inference to the researcher even for models with intractable likelihoods.

We also inform the editor and reviewers that further work based on our manuscript has been recently released as a preprint: "Variational Bayesian Monte Carlo with Noisy Likelihoods" (Acerbi, 2020, *arXiv preprint*). In that paper, the key features of the IBS estimator derived and demonstrated in our manuscript (unbiasedness, normality, and estimated variance), and the IBS MATLAB toolbox released with our manuscript, are exploited to perform sample-efficient, Gaussian-process based Bayesian inference for models with intractable likelihoods. We are mentioning this because it is already another major application for IBS (briefly mentioned in our manuscript), which further shows the usefulness and high potential impact of the IBS estimator, beyond the example applications to maximum-likelihood estimation that we present here.

In the following pages, we reply in detail to the individual reviewers' comments.

Bas van Opheusden
Luigi Acerbi
Wei Ji Ma

**Comments to the Authors:**

**Reviewer #1: REVIEW OF UNBIASED AND EFFICIENT LOG-LIKELIHOOD ESTIMATION WITH INVERSE BINOMIAL SAMPLING**

**Summary**

**This paper introduces Inverse Binomial Sampling (IBS) as a method to compute (log) likelihoods when exact computation is intractable. It is shown that IBS provides unbiased estimates of the likelihood and that it compares favourably to an alternative fixed sampling procedure.**

**Evaluation**

**I think the IBS procedure is an elegant method to estimate the log likelihood from simulation when analytical computation is infeasible. I have not come across this method explicitly, and I think it deserves to be brought to the attention of researchers. I do find the paper rather long however, and I think the method could be presented and evaluated in a manuscript almost half as long. In addition, I think the paper could be clearer on a number of points, detailed below.**

**Major issues**

**1. I found the paper overly long. As I see it, the main message is (1) IBS offers a method to obtain an unbiased estimate of $\log L = \sum_{i=1}^n \log p_i$ by simulation from a generative model. It also offers a way to match simulation cost to the difficulty of estimating small p_(i). As log p is not a polynomial function, and "Kolmogorov (1950) showed that the only functions f (p) for which the fixed sampling policy with M samples allows an unbiased estimator are polynomials", there is no unbiased estimate of log p under fixed sampling. (2) Focusing on the log likelihood of all data is useful as it allows the central limit theorem to apply. (3) While IBS is unbiased, its variance is higher than that of fixed sampling. This may be a situation where minimum bias is more important than minimum variance. (4) Numerical examples show that the method performs well and is efficient.**

We thank the reviewer for the careful reading of our manuscript, this is indeed a good summary of our main points.

In addition, we also believe that the ability of IBS to provide a calibrated estimator of the variance of the estimate can be quite useful in practice (e.g., to develop adaptive methods, or in combination with heteroskedastic Gaussian Process regression, as we argue in the manuscript).

**I don't think the numerical examples need too much explanation of the underlying experiments or models, and as such the whole paper could probably take about 50% of the current word count and be more focused and more impactful. As much of the theoretical developments are derived as a special case of the more general results in de Groot (1959), the reader could be referred to there for some of the more intricate mathematical details.**

While we agree that a shorter paper would be desirable in theory, in practice all reviews asked us, implicitly or explicitly, to add *more* details and clarifications.
We also note that a large part of the current manuscript is taken by the Appendix, which disappears into a separate Supplementary Material file for PLoS submissions (we had included it together with the main text in our first submission for ease of reference, but for resubmission we have been asked by the editorial team to separate them).

With respect to reducing the space dedicated to the simulated experiments, our stated goal is to introduce and validate the IBS method on concrete problems in a self-contained manner and for a wide audience with various levels of technical expertise. For this reason, we believe that spending some time explaining the example models (and how the data are generated in the experiments) in detail has didactic value.
Also, our mathematical explanations and (re)derivations of some properties of the estimator are substantially simpler than the original de Groot (1959) paper, which provides very general theorems whose immediate application to the case at hand (estimation of log-likelihoods) is far from obvious. We believe there is value and usefulness for the target audience of this manuscript in presenting and discussing these results in a more accessible way.

**2. While the fixed sampling scheme has relatively large problems estimating small probabilities, isn't the opposite true for IBS. Does this method not find it more difficult to distinguish between p = .9 and p = .8 than e.g. p = .05 and p = .15? If so, what does that imply for model selection between two models which are both quite good (e.g. nested models with a small change).**

Perhaps counter-intuitively, this is not the case: IBS is quite good at estimating high probabilities. Not only is IBS unbiased for any probability p, the variance of \hat{L} (estimator of log p) also decreases as p → 1, as shown in Figure 1C in the manuscript. In this limit, log(p) is approximately equal to 1-p (first order Taylor series), hence estimating log(p) is easy for large p.

**3. I think it would be good to more explicitly state that the fixed sampling procedure provides a biased estimate of log p, but an unbiased (and minimum variance) estimator exists (and is simple) for _p_. There are good reasons to focus on the estimation of log p, but these can be stated more strongly (e.g., even if you have unbiased estimates of each p_(i), will you then have an unbiased estimate of ∏_(i)p_(i)?**

Thanks for raising this subtle point, also remarked by Reviewer #3. Given its importance, we included a new section in the revised manuscript ("*Why not an unbiased estimator of the likelihood?*") that directly addresses this point.

In short, while it is true that fixed sampling affords an unbiased estimate of p_(i), and thus an unbiased estimate of ∏_(i)p_(i), the resulting estimator would be horribly multimodal with the main peak being at ∏_(i)p_(i) = 0. This property makes this estimator unusable for all practical purposes (e.g., from maximum-likelihood estimation to Bayesian inference). The key property of the IBS estimator of the log-likelihood is not just that it is unbiased, but that it is very well-behaved (i.e., normally distributed - statistically speaking, it doesn't get better than that).

**4. I did not find Section 4.5 "Bias or variance?" too convincing. Estimation typically involves a bias-variance tradeoff. There are often situations where introducing a little bias is outweighed by the associated reduction in variance. Rather than plotting bias and in Fig 1 and 2, it would be more useful to plot something like the MSE, which incorporates both bias and variance, and discuss the tradeoff in more detail.**

This is a great point, and one that deserves careful discussion.
Plotting the RMSE is complicated by the fact that it depends on another factor (besides *p* and the number of samples), that is the number of trials N. Taking into account the number of trials is absolutely key because bias and standard deviation scale differently with N, a property which becomes crucial in practical applications (as we argue, e.g., in Section 4.5).
However, once we introduce N, it becomes clear that a plot identical to Figure 1 is not particularly meaningful - because trials would not all have the same p; a real dataset and model correspond to a *distribution* of p_i.

Thus, to make meaningful RMSE plots, we took as an example the psychometric model described in Section 5.2, and calculated the distribution of p_i for typical datasets and parameters settings. We then calculated the RMSE in estimating the *total* log-likelihood of a number of randomly generated datasets (sampled from the empirical distribution of p_i) with different number of trials; for different numbers of samples used by the IBS and Fixed estimators.

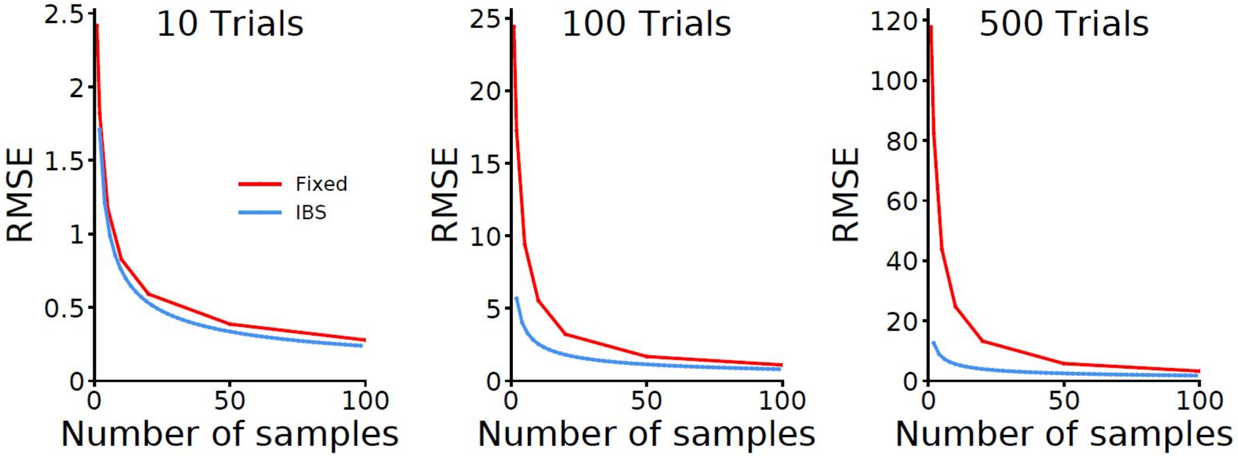


We see in the Figure above that starting from even a handful of trials (N = 10), IBS is consistently better than Fixed, and overwhelmingly so for a moderate number of trials (N >= 100). Considering that there are virtually no studies with less than 10 trials per dataset, and the norm is of the order of a few hundreds trials per dataset, it is fair to say that we expect IBS to consistently outperform Fixed in all practical situations.

We included this Figure in a new section of the Appendix, A.6.

**Both are important, and I think a statement like "Bias can cause researchers to confidently draw false conclusions, variance causes decreased statistical power and lack of confidence." is quite misleading. High variance of estimates can equally lead to false conclusions, if the variance is not properly taken into account in the uncertainty of the results.**

Our main points, and the reason why in Section 4.5 we make a case "against bias", is that:

- There are easy ways to estimate variance (e.g., IBS provides an estimator, and even without that, variance is easy to estimate via bootstrap or repeated sampling), but not easy ways to estimate bias.
- There exist principled methods to account for variance in log-likelihood estimation, such as Gaussian Process regression methods applied to parameter optimization or Bayesian inference.
- Bias, however, cannot be treated properly because it is impossible to know how biased the estimators are, and how the bias changes in different regions of parameter space, or across models, without knowing the true model probabilities.

Still, we completely agree with the reviewer that both bias and variance are important. We better stated our position in the manuscript, by making several edits to the text. Among other changes, we modified the quoted sentence as follows: "*Bias can cause researchers to confidently draw*

*false conclusions, variance, when properly accounted for, causes decreased statistical power and lack of confidence*."

We also added the following paragraph at the end of the section:

"*Finally, while for the sake of argument we structured this section as an opposition between bias and variance, it is not an exact dichotomy and both properties matter, together with other considerations. For example, there are situations in which the researcher may knowingly decide to increase bias to reduce both variance and computational costs. Notably, while this trade-off is easy to achieve with the IBS estimator (see Appendix C.1), there is no similarly easy technique to de-bias a biased estimator.*"

**5. The estimate of the variance of the IBS estimator is derived from a Bayesian procedure. More specifically, "Crucially, Equation 29 shows that we can recover the IBS estimator as the posterior mean of log q given K, under the Haldane prior for q. This interpretation allows us to also define naturally the variance of our estimate for a given K, as the variance of the posterior over log q," I don't see how the (frequentist) variance of an estimator (i.e. the variance of the sampling distribution of the estimates produced by an estimator) equates to the variance of a posterior distribution of a quantity, for which the posterior mean equals the estimator. Especially when this is done under a specific Beta(0,0) prior… I may be missing something, in which case the correspondence could be explained in more detail.**

The variance of the IBS estimator, from a frequentist point of view, is given by Equation 15 in the main text, that is $Var[\hat{L_i}]=L_2(1-p_i)$ where $L_2$ is the dilogarithm. However, this variance is a function of the unknown probabilities $p_i$. One could estimate the variance of $\hat{L_i}$ by first estimating $p_i$ and plugging that into the dilogarithm function, but this is improper and somewhat impractical, so it is not what we suggest to do in the paper.

Instead, an alternative approach of empirical interest is to consider the (posterior) variance of a specific estimate, which can be addressed in a principled way via Bayesian reasoning. First, we find the prior under which the Bayesian estimator equals the IBS estimator (the Haldane prior). Then, we compute the variance *of a given estimate* (which is the quantity of interest), as per Equation 16. Note that this formula differs from Equation 15 (that is, the two approaches are not identical: we are not recovering the frequentist estimator), in that $p_i$ now does not need to be known. While the Bayesian approach is sensible, it does not necessarily guarantee that the resulting variance is calibrated; this is something we validate empirically in Figure 3C.

For clarity, we added the following paragraph in the text:

*"Note that Equations 15 and 16 correspond to slightly different concepts, in that the former represents the variance of the estimator for a known p (from a frequentist point of view), while*

*the latter is the posterior variance of L for a known K (for which there is no frequentist analogue). See also later "Higher-order moments" for further discussion.*"

**Minor issues**

**- "Inverse binomial" is more commonly known as "negative binomial"; this might also result in a more favourable acronym (IBS is a.k.a. irritable bowel syndrome)**

Thanks for the remark. The "inverse" in "inverse binomial sampling" relates to the fact that this is an "inverse sampling" method. In fact, the term *inverse binomial sampling* has been coined and used before, see for example "Inverse binomial sampling as a basis for estimating negative binomial population densities" (Gerrard and Cook, 1972, *Biometrics*). For this reason, we would stick to the existing name.

**- p8. "can be a scalar of vector" -> "can be a scalar or vector"**

Thanks, we fixed the typo.

**- Equation 1. D is defined as the set of all responses and stimuli. The definition of p(D|θ) in Equation 1 contains the stimuli only on the rhs of the conditional sign, i.e. it is for p(r|s, θ) rather than p(r, s|θ). These are not equivalent.**

Thanks for pointing this out. This is an abuse of notation on our side, that we clarified in the revised text. While formally the dataset D comprises of both stimuli and responses, the likelihood we care about is p(r | s, θ) as we assume (by definition of s) that the stimuli are statistically uninteresting. We also specified in the text that this choice is without loss of generality.

In particular, we added the following paragraph:

"*Note that Equation 1 assumes that the researcher is not interested in statistically modeling the stimuli, which are taken to be given (i.e., on the right-hand side of the conditional probability). This choice is without loss of generality, as any variable of statistical interest can always be relabeled and become an element of the `response' vector. For compactness, from now on we will denote* Pr(r | s, θ) == Pr(D | θ)*, in a slight abuse of notation.*"

**- p11. "For example, models with latent dynamics might be able to generate a full sequence of responses given the stimuli, but it might not be easy (or even possible) to generate the response in a given trial, conditional on a specific sequence of previous responses." Would it be possible to specify an equivalent version of IBS counts how**

**long it takes to sample a whole sequence of responses exactly? This would no doubt take quite some time, but wouldn't this also be an unbiased way to estimate log p?**

Yes, this is possible and would work in theory, but the time it takes is exponential in the size of the data set, so effectively impossible to use in practice unless the number of trials is very small. Still, for clarity we replaced "(or even possible)" with "or computationally tractable".
Incidentally, this is the same reason for which IBS cannot be used in practice to estimate the log marginal likelihood of a dataset, log p(D), as it would need to jointly sample the exact dataset.

**- p13. "in which case the central limit theorem guarantees". There are some conditions for the CLT to hold, such as finite variance. It would be good to check and state that these hold.**

Yes, IBS has uniformly bounded variance, which is a sufficient condition for a variant of the central limit theorem to hold. We address this in more detail in section 4.6 ("higher-order moments") and specifically footnote 3 (now footnote 2). Note that the standard CLT does not apply here since the estimators are not identically distributed.

**- p14. IBS is introduced as a sampling scheme which stops as soon as a single "hit" is observed. But it can also be defined for any number of hits greater than 1, Would other forms be less (or maybe more) useful?**

Using multiple "hits" would work and it can indeed be useful, as demonstrated in the paper.
In our paper, we first construct the basic estimator (with a single hit). Multiple-hits estimators with R hits are completely equivalent to what we call "repeated" estimators in Section 4.4 ("Iterative multi-fidelity"), and those are the ones we also consider in our empirical validations, for varying values of R >= 1.

**- Equation 11 uses an additional constant = 1 to avoid a negative infinite log. But might other choices e.g. .5, or .1, not provide better results?**

Other choices of constant would provide quantitatively but not qualitatively different results.
First, note that we tried an alternative form of the Fixed estimator for one of our example problems, with a constant of 0.5 (Equation 32 and Fig. 14 in the Appendix), finding essentially the same results as the Fixed estimator proposed in the main text. For the revision, we also tried yet another variant of the Fixed estimator with a smaller constant of 0.001, which again performs poorly (see these additional results in the response to Rev #3).
Second, our arguments in Section 3.1 of the paper ("Why fixed sampling cannot be fixed") hold regardless of the exact form of the Fixed estimator.
Finally, more in general, the choice of regularization in the expression for the Fixed estimator roughly corresponds to a "prior" on $p_i$, though not in a strict sense. If that prior is well matched to the dataset and model, fixed sampling can do reasonably well. However, in practice it is rare that all trials are i.i.d. (e.g., stimuli will differ), meaning that a constant regularization is unlikely to

work well for all trials. Moreover, knowing what regularization to use on a trial-by-trial basis is only possible if one knows the true probabilities p_i for each trial.

**- p22. "We saw earlier that for fixed-sampling estimators to be approximately unbiased, one needs at least 1/p_(i)samples, and IBS does exactly that." Surely, that only holds in expectation.**

Correct, thanks for noticing. We clarified it in the text.

**- p23. "We note that fixed sampling eventually saturates the information inequality". This is better than IBS achieves. But more importantly, when does it achieve the equivalent of IBS (e.g. a close 30% to optimum)?**

It depends on p, therefore it is difficult to give a simple answer. Also, it might not be a particularly meaningful comparison. To compare the performance of IBS and Fixed, we suggest to look at the RMSE plots we made above.

**- p23. An estimator for the variance of the estimator of IBS is derived, but how good is this estimator? Is it unbiased, efficient?**

These are great questions and we leave a mathematical proof as an open problem for future work. In our attempts so far, doing these calculations analytically seems intractable as the trigamma function does not behave "nicely". However, as noted before, a detailed mathematical analysis goes beyond the scope of our work. In our paper, we address the quality of the variance estimator numerically, by providing a calibration plot (Figure 3C).

**- p25. "fixed sampling can have slightly lower variance." How is "slightly" defined. Personally, it looks to me that it can achieve substantially less variance.**

In order to appropriately compare the variance of Fixed sampling and IBS, we also need to take into account the number of samples. To do so, in Figure 13 we plot the standard deviation of the estimator times the (square root of) the number of samples for both estimators, as a function of true p. Fixed sampling at its best will approximate the black line, while IBS will always be at the blue line. We can see that in this plot, for values of $p > 0.1$, the blue line is within 25-30% of the black line. We agree that it is unclear whether this is "slightly" lower, so we removed the adjective from the text and just say "can have lower variance".

In practice, for a meaningful comparison between the two estimators, we would refer the reader to the new RMSE plots (see above).

**- p25. "Basic rules of probability imply…" Not sure what these "basic" rules are.. I assume those applying to expected values of sums of independent variables?**

Correct, we specified it better in the revised text.

**- p26. "By contrast, no optimization algorithm can handle bias.". This might be true, but it might also not matter if the bias doesn't affect where the maximum is.**

That's correct if the bias is constant, or e.g. it follows some monotonic relation with respect to the distance from the true optimum, among other forms. In other words, yes, surely one can construct *theoretical* scenarios in which the bias is not constant, but still irrelevant for the purpose of maximum-likelihood estimation.

However, we see that in common empirical scenarios the bias can and does affect the location of the maximum quite strongly. One such case emerges when estimating parameters which relate to the amount of noise in a model, such as the lapse rate and measurement noise in the two perceptual models. Such parameters are consistently underestimated with Fixed sampling because the regularization constant of the sampling algorithm approximately acts as if the model already had an implicit lapse rate (that is, no trial can have a log likelihood less than a threshold value). For this reason, there is "less need" to explain away variability in the data using noise, and noise parameters tend to be underestimated.

Finally, we specify in the text that the argument is not limited to maximum-likelihood estimation. For example, posteriors inferred via approximate or full Bayesian inference would be strongly affected by bias in the log-likelihood (unless the bias is constant everywhere, which is unlikely).

**- Figure 3A. This distribution looks clearly skewed, so I don't agree with the statement "By comparing the histogram of z-scores with a standard normal in Figure 3A, we see that the total number of samples is approximately normal."**

Yes, there is some skew (that is why we wrote that the shape is "approximately" normal). Still, to be completely clear, we added "*with some residual skew*."
For positive-only variables (such as number of samples), the central limit theorem still holds, but it might take a large N for the skew to disappear completely, and this residue is what we observe here. Still, for most practical purposes, a normal distribution is a good approximation of the distribution of the number of samples.

In particular, one situation we have in mind is when a researcher is re-evaluating a parameter setting multiple times (e.g., to evaluate the log-likelihood at the returned solution with high precision). In this circumstance, one can get a quick estimate of the total required computation time by running IBS a few times and estimating mean and variance. Since the distribution is approximately normal, the expected running time and its variance are easy to estimate; one should not expect arbitrarily large deviations.

**- p29. "If such estimate is correctly calibrated," -> "If such an estimate is correctly calibrated,"**

**- p33. “regardless of of the number” -> “regardless of the number”**

Thanks for spotting these typos, fixed.

**- Figure 5. “For fixed sampling and IBS, we plot mean and standard error as a function of the (average) number of samples used.” But doesn’t the number of samples for IBS depend on p? I don’t think there is a clearly interpretable comparison between the expected IBS samples and the M samples for fixed sampling.**

Yes, the number of IBS samples depends on p, which is a function of the data set and model used. We use the same data sets for both IBS and fixed sampling, which means these comparisons are fair.

**- “Interestingly, maximum-likelihood estimation via the ‘exact’ method provides biased estimates of η when the noise is high.” While it makes sense that “sensory noise and lapse become empirically non-identifiable for large η”, why doesn’t this hold for sampling approximations?**

This is a great question and we addressed it briefly in a paragraph of the text as it is not a main point of the paper, but we can expand on it here.

First, note that any stochastic optimization method implicitly provides a (weak) *effective* prior over the parameter space, induced by the heuristics and assumptions used to guide the search and judge where the optimum is located in light of a sequence of noisy function observations. For example, most optimization methods explicitly or implicitly make assumptions about smoothness of the function landscape. This is particularly true for derivative-free optimization methods, where a (noisy) gradient is unavailable; for a review, see for example “Derivative-free optimization: a review of algorithms and comparison of software implementations” (Rios & Sahinidis, 2012, *Journal of Global Optimization*).
Second, a lack of parameter identifiability is “solved” in the presence of a regularizer or prior (although the regularized solution does not necessarily pinpoint to the true value).

What is happening here is that the implicit prior induced by our optimization method (more specifically, the Gaussian process assumptions adopted by the BADS optimizer) turns out to break lack of identifiability in a way which happens to be advantageous for recovering this specific parameter. However, we absolutely do not claim that this would be generally the case. We explained this issue in the text as follows (adding in the revision the sentence about search biases):

“*IBS and fixed sampling perform seemingly better here [than the exact method] because the interaction between a ridge (or flat region) in the true likelihood landscape and noisy estimates thereof depend on specifics of the problem and of the optimization procedure (e.g., implicit or explicit search biases adopted by the optimizer, such as smoothness assumptions). For these*

*particular settings of θ_true, IBS and fixed sampling perform better at recovering η than the ‘exact’ method, but it does not necessarily hold true in general.*”

**- p44. “The results in Figure 10 show that IBS, even with only a few repeats, is able” How many repeats?**

How many repeats are needed to obtain “good” ML solutions via maximum-likelihood estimation depends on how close to the true maximum log-likelihood one would like to get (and specifics of the problem). For our examples, R=1 (i.e., running IBS once) is enough for both orientation discrimination and change localization problems to get within 2 points of the true maximum log-likelihood, on average. To get within 1 point, one needs R=2 for the orientation discrimination model and R=3 for change localization.

Note that this value of R represents the number of repeats used for each log-likelihood evaluation *during the optimization process*. To report the actual log-likelihood at the returned solution, we assume that the researcher will then re-evaluate the log-likelihood a large number of times to achieve the desired precision in the final estimate. The point is clarified in the text as follows:

“*Note that our analyses of the loss are based on the `exact' log-likelihood values evaluated at the solution returned by the optimization procedure. In practice, we would not have access to the `exact' log-likelihood at the solution; but its value can be estimated up to the desired precision with* <u>*IBS*</u>*, by taking multiple repeats at the returned solution.*”

**- Figure 10. “Lines are mean and standard error” Personally, I would find it more informative to depict quantiles of the distribution of estimates.**

Thanks for the suggestion. We show below the plot with median and interquartile range.

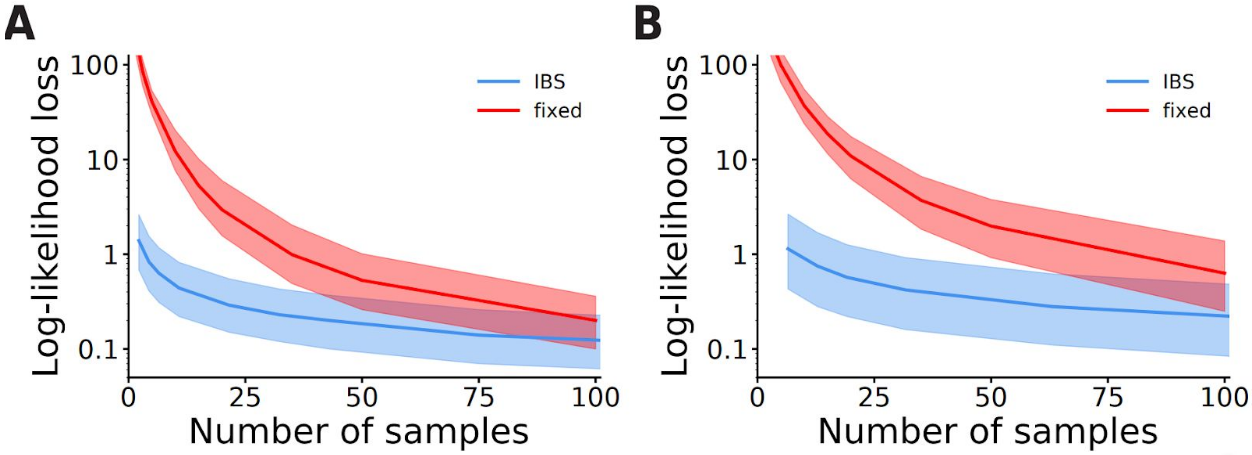

It turns out that the plot with mean and standard deviation (in log scale) is similar (see below; the larger shaded area is because +/- 1 SD contains ~68% probability, while the IQR 50%). For consistency with the rest of the paper, we used this in the manuscript.

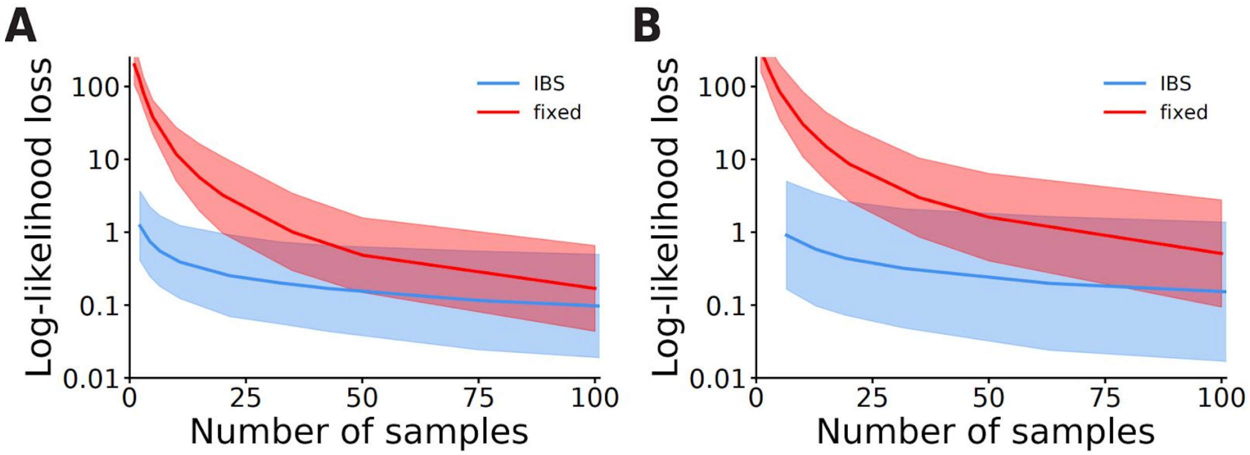


**- “whereas fixed sampling remains severely biased even with large number of samples”. What it “severe” here? In general, such hyperboles should be avoided (e.g. also on p46 “severely” and “close to useless”)**

Throughout the manuscript we used “severe” to indicate *substantial* effects with practical consequences (as opposed to, e.g., “statistically significant” but small effects). In this specific case, by “severe” we mean much larger than ~1 point of log-likelihood loss, which we deem a reasonable error threshold (we explained it in the revised text).

More in general, we agree these terms should be used sparingly, but we do not think we are exaggerating in this specific case. The log-likelihood loss of fixed sampling is about an order of magnitude higher than that of IBS. In parameter estimation with the different models, we also see that fixed sampling can underestimate lapse rates by a factor of 2, and measurement noise parameters by eta- \hat{eta}~1, which means sigma = exp(eta) is sometimes underestimated by a factor of exp(1) = 2.72.

To be more clear, we substituted “severe” with “substantial” in most parts of the text (as “substantial” is more commonly associated to denote a large effect size). We also replaced “*close to useless*” with “*hardly usable in practice*”.

**- p47. “We developed inverse binomial sampling for log-likelihood estimation of likelihood-free models”. That seems contradictory. How can you estimate the log likelihood when there is no likelihood? Models where the likelihood is infeasible to compute would seem more accurate.**

Correct - we mean that the likelihood is not available to the researcher, not that the likelihood does not exist at all. This is a “punchy” abuse of language nodding to “likelihood-free” inference or “likelihood-free” methods, the common way to denote the problem of performing likelihood-based inference in the absence of an explicit likelihood function. See for example “ABC likelihood-free methods for model choice in Gibbs random fields” (Grelaud et al., 2009, *Bayesian Analysis*). We modified the sentence to “*We developed inverse binomial sampling for log-likelihood estimation of models with intractable likelihoods*”.

**- p56. “First, we can use the inverse binomial sampling policy to estimate any analytic function of p.” Why?**

Because one can choose the a_k in Equation 26 to be the coefficients of the Taylor expansion of f(p) (that is, the definition of an analytic function).

**- p59. “Therefore, the standard deviation in IBS is close (within 30%) to its theoretical minimum.” I don’t see how this follows from the preceding.**

Thanks, we clarified the text by adding: “*Applying this argument to f(p) = log(p), we find that* [...]”.

**- p64. “To avoid wasting computations on ‘bad’ parameter settings, for IBS we used the ‘early stopping threshold’ technique described in Section C.1” Doesn’t this reduce the effective number of samples for the IBS, in which case this should be clearly specified in the comparisons of samples between IBS and fixed sampling.**

We added a note in the “Procedure” section of the Results (formerly Section 5.1): “*To prevent IBS from `hanging' on particularly bad parameter vectors, we adopt the `early stopping threshold' technique described in Appendix C.1*.” However, we would also like to remark that the early stopping threshold has little impact on our analyses and it’s not giving IBS an unfair advantage.

First, note that fixed sampling has constant time cost for each parameter vector tried, but IBS takes more time for worse vectors, which are less useful for optimization. So, if anything, IBS is penalized for sampling in bad regions, while Fixed sampling is relatively unaffected. The threshold is introduced in principle as a simple method to ameliorate this issue of IBS.

Second, the early stopping threshold we chose is rarely reached during our experiments. For example, by rerunning our analyses again while counting each time the threshold was hit (a metric we did not record in our previous analyses), we found out that in the psychometric model the early stopping threshold was reached in only 2% of evaluations.
The reason is that a parameter setting (or model) needs to be *particularly* bad for the log-likelihood to be below the chosen threshold (chance level). Still, an early stopping threshold

is useful to have to prevent IBS from “hanging” for a very long time on those few cases in which a particularly bad parameter vector has been picked.

**Reviewer #2: This paper presents an inverse binomial sampling method for estimating the log-likelihood of simulation-based models given an experimental data set. The authors show that estimators from IBS are uniformly unbiased, their variance is uniformly bounded, and a calibrated estimator for the variance is given. But I have the following comments.**

**1. When there is not a likelihood function, a nonparametric method such as empirical likelihood has been developed to estimate the probability p_i under the restriction and it is easily implemented. For the current method, the authors did not consider the restriction among p_i. Generally, should consider it. In particular, for fixed method, one can regard the considered case as a multinomial distribution. How is the result?**

We thank the reviewer for their careful reading of our manuscript and for their interesting comments.

The empirical likelihood method suggested by the reviewer is used to non-parametrically characterize a distribution of i.i.d. samples under some chosen restrictions or constraints (e.g., on the moments of the distribution), in an approach similar to maximum-entropy. However, the main objections to using the empirical likelihood for our purposes are:

1. The data points from different trials are not necessarily i.i.d. (nor are necessarily easily transformed to an i.i.d. sequence).
2. We assume no constraints on the distribution of data points. In particular, we want to be able to calculate the log-likelihood for arbitrary models with often meaningful parameters, as opposed to “descriptive” parameters used by the empirical likelihood method, represented by (often simple) functionals of the empirical distribution, such as the moments.

For these reasons, while interesting for other forms of statistical inference, and there are situations in which the empirical likelihood method can be used as an alternative to likelihood-free inference (see e.g. “Bayesian computation via empirical likelihood”, Mengersen et al., 2013, *PNAS*), we do not believe the empirical likelihood method to be an answer to the *general* problem of estimating log-likelihoods of an entire data set when only a model simulator is available.

Finally, we remind the reviewer that our objective of estimating log-likelihoods is much more general than that of estimating parameters. While in this manuscript, for simplicity and didactic purposes, we demonstrate the properties of IBS in the context of maximum-likelihood estimation, the IBS estimator per se unlocks the entire toolbox of *likelihood-based inference*.

**On the other hand, we may not be care the bias rather than variance, for example, the biased estimator.**

We discuss our rationale for an unbiased, normally-distributed estimator of the log-likelihood in Sections 3.2 ("Why not an unbiased estimator of the likelihood?") and 4.5 ("Bias or variance?"). See also our previous response to Reviewer #1.

**2. Page 18, section 3.1, line 4: choosing M requires knowledge of p_i on each trial. However, the authors' main purpose is to estimate p_i. Why?**

Our argument here is that choosing an appropriate M for a Fixed sampling method so as to meaningfully reduce the bias would require knowledge of p_i in each trial, but crucially the p_i are unknown.
Our main goal is to estimate log p_i for each trial, so as to compute the total log-likelihood, as per likelihood-based inference. The log-likelihood represents a principled quantity to judge goodness-of-fit of a model. In this paper, we focus on maximum-likelihood estimation, although a similar approach would work for Bayesian inference.

**3. Algorithm 1: Generally, theta is unknown. How to sample from model (M,theta,..). Also, how to estimate theta in the considered model.**

IBS provides a technique to estimate the log-likelihood of a model and data set for a given parameter setting θ. For maximum-likelihood estimation, candidate θ's will be provided by the optimization algorithm. In our case, we used Bayesian Adaptive Direct Search, or BADS; but any derivative-free stochastic optimizer could be used as well.

We clarified this point in the text as follows:
"*Finally, one might ask how to choose the θ to evaluate in Algorithm 1 in the first place. IBS is agnostic of how candidate θ are proposed. Most often, the function that implements Algorithm 1 will be passed to a chosen optimization or inference algorithm, and the job of proposing θ will be taken care of by the chosen method. For maximum-likelihood estimation, we recommend derivative-free methods that deal effectively and robustly with noisy evaluations, such as Bayesian Adaptive Direct Search (BADS; Acerbi and Ma, 2017) or noise-robust CMA-ES (Hansen et al., 2003, 2008).*"

**4. Eq. (16): Is it the variance or the estimated variance of a specific IBS estimator?**

Thanks for the question, it is something we should have explained better. Equation 16 is a Bayesian posterior variance for a specific estimate of the log-likelihood. We added a clarification to this part in the revised text.

**5. Section 5.1: what is theta? How to estimate it?**

As mentioned above, candidate θ's will be provided by the optimization algorithm, and IBS will estimate their log-likelihood as per Algorithm 1. The returned optimum is a candidate maximum-likelihood solution (to compute the MLE, we take the best out of four or eight optimization runs, as explained in the paper).

**6. Page 33, the second paragraph: It is unclear how to estimate eta and gamma.**

$\eta$ and $\gamma$ are two model parameters (part of the parameter vector $\theta$). $\theta$ is estimated via maximum-likelihood, as explained above.

**7. Page 35, the second paragraph: when the patches are from different distributions, how is the result?**

We have not simulated data with change localization with other distributions; it is an interesting modeling and empirical question, but beyond the example we are considering here.

**In particular, when there are outliers in the considered dataset, how is the result? The proposed method is robust.**

The log-likelihood estimates of IBS are always unbiased, independently of outliers. In practice, when used to perform maximum-likelihood estimation, IBS is robust to outliers and heavy-tailed distributions as much as the combination of model, data and optimization method can be (depending on parameters such as noise and lapse rate).
Conversely, the Fixed approach will generally be worse. For example, as mentioned in an answer before, Fixed sampling will generally be worse at inferring noise and lapse parameters.

**Also, when the data are from the heavy-tailed distribution, how is the result?**

To answer this question and explicitly show that IBS can be used also with data from a heavy-tailed distribution, we simulated data from a Student's *t*-distribution with $v = 3$ degrees of freedom. This is an example of a heavy-tailed distribution (we chose $v = 3$ such that the mean and variance are finite).

Specifically, we generated datasets with $N$ = 100 data points each, for different values of the location and scale parameters, respectively μ and σ, of a Student's *t*-distribution (100 datasets per parameter setting). For each dataset, we then recovered the location and scale parameter with maximum-likelihood estimation via the exact (analytical) likelihood, and via IBS. Note that since IBS only works with discrete data, we binned the samples in a fine grid from [-10, 10] with step size 0.1 (the bounds are chosen to contain almost all the probability mass, for the considered values of μ and σ). Like in all examples in the paper, we parameterize σ as η = log σ.

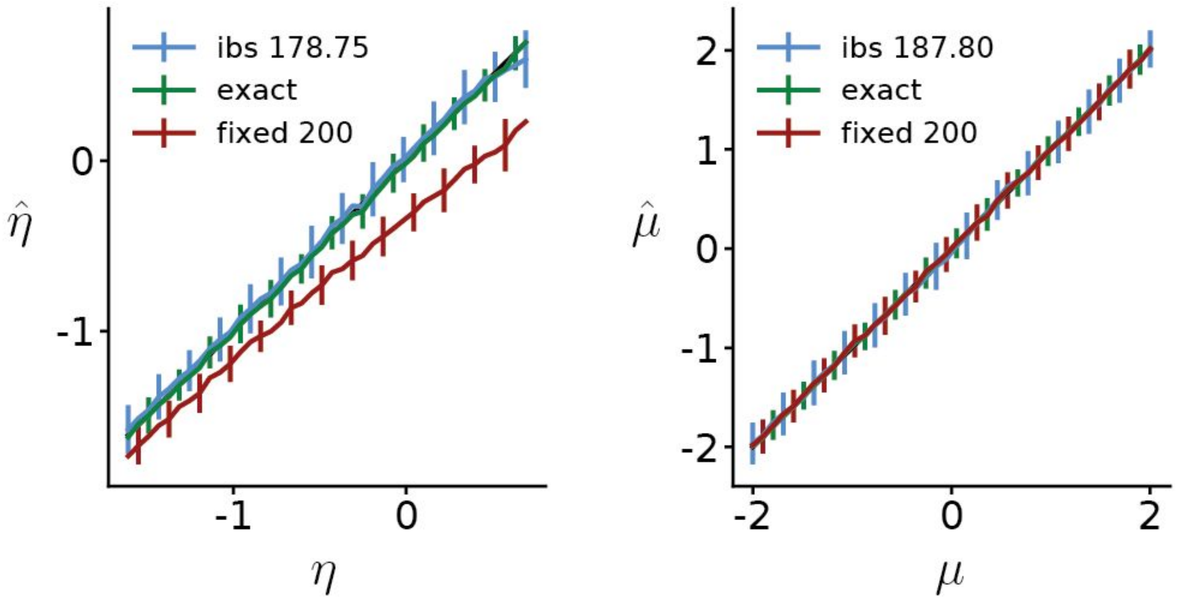


We can see in the Figure above that all methods are able to recover the location parameter μ, but regarding the noise η, IBS is able to recover the true parameter whereas fixed sampling fails.

**Reviewer #3: The current MS focuses on a common situation where complex computational models are studied based on maximal likelihood (ML) estimation but the likelihood function is unavailable in analytical form or even in a handy numerical form.**
**In such cases one must resort to simulations for estimating the likelihood. The authors show that a commonly used method that estimates likelihood based on a "fixed sample size" simulations has important limitation. For example, it can lead to wrong scientific conclusions in the form of false model comparison inferences and can result in severe biases in estimated parameters. The authors argue the main reason for this is that this method provides biased estimates of log-likelihoods. As an alternative, they present a method based on "inverse binomial sampling" (IBS) that returns non biased log-likelihood estimates. The authors show that IBS has several desirable statistical**

**properties and that in 3 studied examples, it performs better (in parameter-estimation) than the "fixed" sampling method.**

We thank the reviewer for the thoughtful reading of our manuscript.

We would like to stress one important detail: as the reviewer points out, our manuscript focuses for simplicity on maximum-likelihood estimation (MLE) as a practical *application* of IBS, but IBS per se is a general technique to estimate log-likelihoods in an unbiased and efficient way when the likelihood function is unavailable; and this is what our paper is about. We describe in the Discussion several alternative applications, such as for (approximate) Bayesian inference.

Incidentally, as mentioned in the introduction of our response to the reviewers, a separate preprint recently posted on arXiv builds on our work, applying IBS (among other techniques) to approximate Bayesian inference (Acerbi, 2020, *arXiv preprint*).

**I think this is a timely, topical and excellent study that will be relevant for a wide community of modellers across diverse scientific domains and fields. The authors highlight an unfamiliar (or perhaps relatively ignored) method of ML estimation in complex situation, which can improve the way that the community performs model comparisons and parameter estimation. The authors provide a both theoretical arguments and illuminating simulation studies to convince that IBS has several important advantages over the commonly used fixed-sample size method. However, I also have a few concerns that I think must be addressed before the paper can be published. I think that in some cases the connections between the theoretical arguments and the simulations are not sufficiently established and clear. Below, some thoughts and suggestions for improvement.**

**1) The main argument in sections 2-4 of the paper is that whereas the "fixed" method provides biased estimates of likelihood the IBS provides unbiased estimates. Critically, however, while this is true for any given parameters set it is important to explain to readers that the IBS method will not recover the "true" maximal likelihood value. Suppose that we find the best fitting parameters for some model based on IBS, executing algorithm 1 and using some search (or optimiser) over the parameter-space. Suppose that we can then query an oracle about the true log-likelihood for these best-fitting parameters (i.e., re-calculate the likelihood with infinite precision). Because the parameter-set we found are not the true ML parameters then the likelihood we will obtain must be smaller than the true ML value! Additionally, if we simply rely on the maximal log-likelihood value that was obtained while the search in the parameters space was executed, then we also get biased estimates of ML (because the log likelihood estimate is conditional on being better that other estimates for other parameter-sets; I think it is important to explain this to readers). I think it is critical that the authors discuss these issues as I am worried that some readers may have a false impression that because IBS**

**provides unbiased estimates of the log-likelihood function it can also calculate accurately the true maximal log-likelihood value (when in fact it will necessarily underestimate this value).**

This is absolutely correct, and one of the reasons why we included Figure 10 in the main text. The Figure shows that the log-likelihood *loss* is always positive, in that the exact value of the found maximum log-likelihood ("exact" as in evaluated through an oracle) is always less than the exact value of the true maximum log-likelihood. Still, we agree with the reviewer that this is an important point and we thus added several comments to the main text (see below).

Note that with a few repeats (R=3-5), the log-likelihood found by IBS differs by less than 1 point (added across the whole dataset) from the true maximum, after optimization. Given that model comparisons usually rely on differences between models of at least 5-10 points, and often much larger, an error of up to 1 point is tolerable in most circumstances.

**Because IBS cannot provide an accurate value of the ML it can also lead to false conclusions (e.g., in model comparison based on BIC where the maximal log-likelihood is plugged). For such uses, the issue of ultimate importance when comparing between the fixed method and IBS, is which method estimates more accurately the maximal log-likelihood value and not whether likelihood is estimated with or without bias for each parameter-set.**

Indeed, IBS only provides an unbiased estimate of the log-likelihood for a given parameter setting; there is no claim that it provides an unbiased estimate of the maximum log-likelihood. However, we do claim and provide evidence that using IBS, by being unbiased and normally distributed, helps find better solutions (that is, solutions that actually have higher log-likelihoods, and closer to the ground truth), as shown in Figure 10.

**It is important to clarify these issues. These issues are somewhat implied in section 5.5 but I think this is too late in the paper and that even in this section, these issue is not discussed explicitly enough.**

We agree with the reviewer that these are important issues and this is why we wrote Section 5.5 in the first place. We expanded our treatment in Section 5.5 ("Log-likelihood loss") with the following explanation:

"*Finally, the results in Figure 10 also display clearly that, while IBS is unbiased in estimating the log-likelihood* for a given parameter setting *$\theta$, the maximum-likelihood procedure per se will have some error. Due to estimation noise and specific features of the data, model, and stochastic optimization method at hand, the returned solution will rarely be the true maximum-likelihood solution, and thus, by definition, the value of the log-likelihood at the*

*solution will* underestimate *the true value of the maximum log-likelihood. Still, Figure 10 shows that the underestimation error, at least in the IBS case, tends to be acceptable, as opposed to the large errors obtained with the Fixed estimator*."

In a new paragraph at the beginning of the Results section, we further point this issue out with the sentence: "*Similarly, it is unclear how bias and variance in individual log-likelihood estimate will affect the estimate of the* maximum *log-likelihood, often used for model selection (e.g., unbiased estimates of the log-likelihood do not guarantee an error-free estimate of the maximum log-likelihood, which is affected by other factors; see later 'Log-likelihood loss').*"

**2) Relatedly, in section 5 the authors compare the methods (fixed vs. IBS) in their ability to recover model-parameters. I believe, there should be some preparatory discussion about the relationship between concepts of accuracy of parameter estimation (sec 5) and concepts that pertain to accuracy in likelihood estimation that were introduced in the earlier sections. How are biases (and variances) in likelihood calculation related to biases (and variances) of estimators of model parameters?**

Thanks for the suggestion. We added the following paragraph of explanation at the beginning of Section 5 (now "Results").

"*The rationale for our numerical experiments is that, previously, we have analyzed fixed sampling and IBS in terms of the bias in their log-likelihood estimates for individual parameter vectors. However, these log-likelihood estimators are often used as elements of a more complex statistical procedure, such as maximum-likelihood estimation. It is plausible that biases in log-likelihood estimates will lead to biases in parameter estimates obtained by maximizing the log-likelihood, but the exact relationship between those biases, and the role of variance in optimization is not immediate. Similarly, it is unclear how bias and variance in individual log-likelihood estimate will affect the estimate of the* maximum *log-likelihood, often used for model selection (e.g., unbiased estimates of the log-likelihood do not guarantee an error-free estimate of the maximum log-likelihood, which is affected by other factors; see Section 5.5).*
*Therefore, we conduct an empirical study showing that, in practice, IBS leads to more accurate parameter and maximum log-likelihood estimates than fixed sampling, given the same budget of computational resources.*"

**3) Divergence elimination. The simulation studies the authors present for the "fixed" method focus on eq. 11 or some other ways to eliminate "divergence". This leaves open the question if such "divergence avoidance" methods somehow work against this method. Another possibility that should be examined is to use eq. 10 and simply return a value of minus-infinity (for log-likelihood) if ever m=0 for any of the trials. This will simply mean that this parameter set is excluded from consideration as the ML parameters. We need to see how well this method performs.**

First of all, we note that the manuscript is about comparing methods to evaluate log-likelihoods (in particular, IBS vs. Fixed sampling, a commonly used approach).
The suggested solution, however, implicitly tampers with the optimization procedure, which is not something this paper is focused on (see also a later answer). Point is, there is no “assumptions-free” way to “exclude a parameter set from consideration as the ML parameter [during optimization]”: this will depend heavily on the underlying assumptions or heuristics of the chosen optimization algorithm. As a very practical remark, many optimization algorithms do not support non-finite function outputs such as Inf or NaN (e.g., our chosen optimizer does not), so there might be no way to properly tell the optimizer to exclude a parameter set. Finally, even if this was possible, the suggested approach *would still introduce biases* (by ignoring divergent estimates).

Having said this, in principle the method proposed here by the reviewer is equivalent to replacing the estimate when m=0 with log(epsilon/M), for epsilon → 0. Thus, in practice, we can approximate this approach by using an epsilon small enough such that the estimated log-likelihood will be arbitrarily low, and ignored for all practical purposes (although not *too* low, to avoid numerical instabilities).
In the Appendix, we already considered the case epsilon = 0.5, finding no qualitative differences with the divergence elimination solution adopted in the main text. To simulate the case epsilon → 0, we also ran a new set of analyses with epsilon = 0.001. Unsurprisingly, also this approach performs poorly (see Figure in the next page; the format is the same as Figure 5 in the main text, but we used here the alternative Fixed estimator).

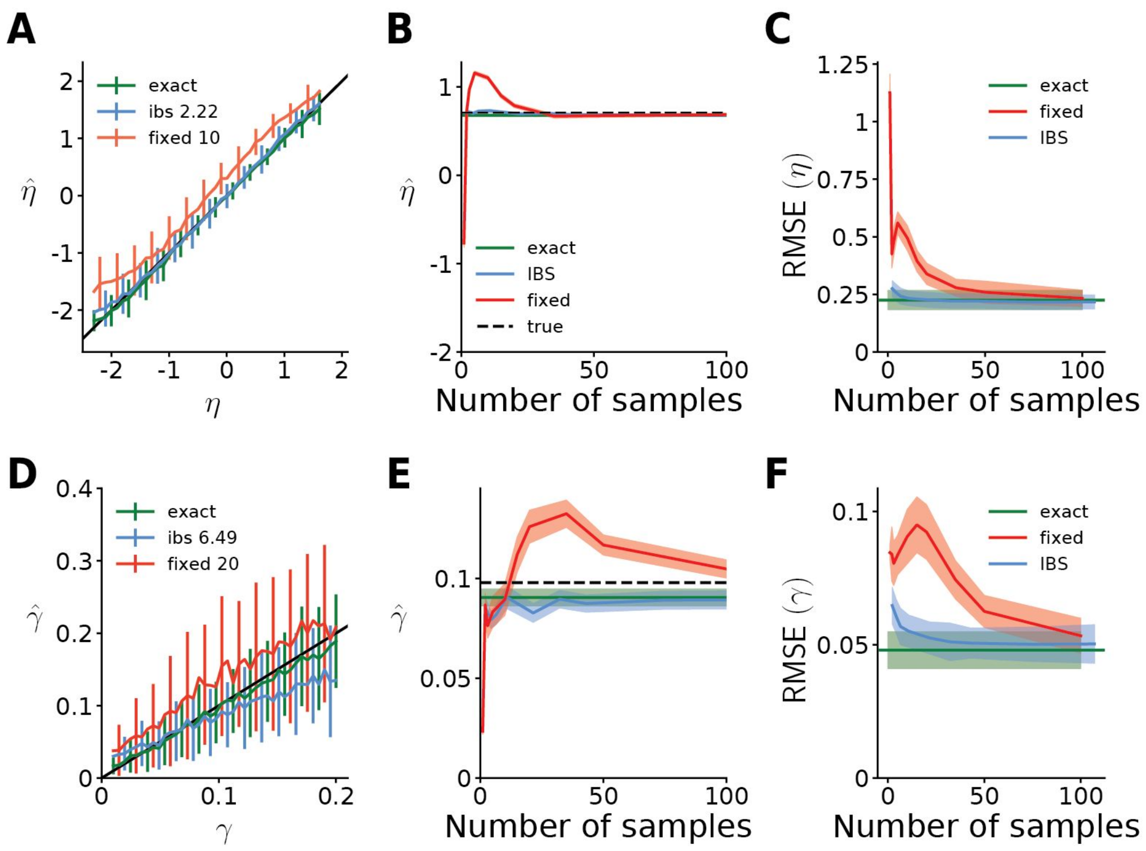


In conclusion, we would like to stress again that *any* attempt to “fix” the divergences will result in introducing biases, as we discussed in several points of the paper.

**4) One issue that is unclear is whether the IBS method performs better because it estimates log(p) in a non-biased way or due to some other reason (e.g., allocating sampling resources more efficiently). Let me elaborate.**
**At the end of p.12 the authors argue that combining estimates of single trial p_i into the product of all pi’s is non-trivial. Why? Can’t we just multiply the independent single-trial estimates to obtain a non-biased estimate of the product? I understand that it is practical to work with logarithms due to numerical issues with performing calculations on computers. But in principle, if we could perform infinite precision-calculations then for finding ML parameters, there would be no advantage is using logarithms- right? In fact, the problems of maximising the product and the sum-likelihoods are mathematically equivalent.**

These are all good point and were raised in a similar form by Reviewer #1. We clarified this very important issue in the revised paper, by including a new section in the revised manuscript (Section 3.2 “Why not an unbiased estimator of the likelihood?”) that directly addresses this point.

As we wrote earlier, while it is true that fixed sampling affords an unbiased estimate of $p_{(i)}$, and thus an unbiased estimate of $\prod_{(i)} p_{(i)}$, the resulting estimator would be horribly multimodal with the main peak being at $\prod_{(i)} p_{(i)} = 0$. This property makes this estimator unusable for all practical purposes (e.g., from maximum-likelihood estimation to Bayesian inference). The key property of the IBS estimator of the log-likelihood is not just that it is unbiased, but that it is very well-behaved (i.e., normally distributed - statistically speaking, it doesn't get better than that).

**This leads to the following question. If we take the exponent of the estimate in eq.10 we get a non-biased estimate of $p_i$ – right?**

Correct.

**What happens if we take an exponent of the IBS estimate (eq.14)? Is this a non-biased estimate of $p_i$ or is it biased?**

It is biased.

**In case it is biased it is unclear why the IBS should a-priori be expected to perform better in estimating parameters. After all, the fixed method (based on eq. 10) estimates $p_i$ with no bias whereas the IBS method estimates $\log(p_i)$ with no bias. Why is it an advantage to estimate $\log(p)$ rather than $p$ with no bias?**

Because our goal is to estimate the log-likelihood (or, equivalently, the likelihood) *of the entire data set*, not of individual trials. It is easy to go from unbiased estimates of the log-likelihood of each trial to an unbiased estimate of the total log-likelihood which is also well-behaved (normally distributed). On the contrary, if we only have unbiased estimates of the likelihood, we can obtain an unbiased estimate of the total likelihood, but it will be generally horribly behaved (e.g., multimodal with a primary mode at 0, which is completely uninformative).

Since parameter estimation requires optimization (for maximum-likelihood estimation) or some form of posterior sampling/approximation (for approximate Bayesian inference), there is huge difference in practice in doing so with a well-behaved smooth surface with Gaussian noise vs. a "Swiss cheese" surface with missing points (the 0s) and multimodal noise.

Importantly, the choice of representation matters! Optimization or approximate inference are not magical black-box oracle devices (as they are often naively assumed to be by practitioners), but they work only through regularity assumptions about the surface being optimized/sampled; the simpler it is, the better the results. Without these assumptions, no method would work better than random search (see also the "No free lunch" theorem for optimization).

**This leads in turn to the following question: Is the IBS method really better because it estimates log(p) with no bias or due to some other reason (e.g., it allocates resources more efficiently?).**

While this is an interesting question, it is hard to disentangle the two aspects of IBS as the derivation of the unbiasedness of IBS also shows that \hat{L}_ibs is the unique estimator which is uniformly unbiased: there are no alternatives if one wants to be unbiased. In other words, we cannot test another unbiased estimator with different properties (e.g., no resource allocation); these aspects are entangled. Our informed intuition is that unbiasedness and normality of the estimator are the two key properties (more so than the efficiency, which is a nice addition).

**Relatedly, can we use IBS to find non-biased estimates of p and then take their logarithm as estimates of log(p)? Would this method work better or worse? And what if we use for IBS the estimation p=1/K or log(p)= -log(K). How would this perform?**

Yes, one could do that, but it is unclear why any of these estimators would be useful. As mentioned above, \hat{L}_ibs is the unique estimator for log(p) which is uniformly unbiased. If we were to use any other function g(K), we would not obtain an unbiased estimate of log(p), but that of a different function, namely the Taylor series in 1-p with coefficients g(K) - g(K-1), as shown in equation 26. Clearly, there is no limit to the alternative *biased* estimators we could try, but unless one is trying to estimate a different function than log(p), we see no particular reason to explore such estimators.

**5) In the discussion of bias and variance (section 4.5.) I think point 5 is unclear. It seems important to consider and explain the issue that if variance is high, then the best-fitting parameters may be very far from the true ML parameters and hence the calculation of the maximal-log likelihood can be very distorted. Thus, high variances can also lead to distorted conclusions (e.g., in model selection). Additionally, cannot high variances in estimating likelihood lead to biases in parameter estimation (once more leading to false conclusions)? So a distinction according to which bias leads to false conclusions whereas variance leads to reduced power seems over-simplified.**

Our main point is that variance, if known, can be at least partially accounted for (for example, the optimizer we are using is able to handle part of it via Gaussian Process regression), whereas bias is by definition unknown. However, we agree that our original presentation was oversimplifying the issue, and we modified the section to better discuss the matter.

**6) In many instances the author focus on the “average case” of using IBS (e.g., average number of samples). They use such consideration to illustrate efficiency. However, many resource limitation may effectively mean that modellers will be interested in “worse case” or at least a “realistic bad case”. Here there is a risk that when p is very low IBS**

**will take too long. In fact, where the fixed method is severely biased (p tend to 0) the IBS takes long to calculate (number of samples tends to infinity). It is important to consider this issue and discuss its implications.**

This issue is discussed in the main text at the end of Discussion, “avoiding infinite loops”. In practice, as simple solutions we recommend to implement a lapse rate in the model, and to introduce an early-stopping threshold. The threshold we discuss in the paper is fixed (e.g., equal to chance level). More sophisticated adaptive approaches could be used, which we leave as a topic for future work.

**The authors describe in an appendix a way to avoid this problem by stopping sampling early. I think this method is also implemented into the simulation- right?**

Correct.

**However, “early stopping” methods can be used in the “fixed” approach as well (e.g., if after calculating p for a subset of trials it is already evident that the current parameter-vector cannot be the ML there is no use to continue to calculate likelihood for other trials).**

This is an interesting idea, although not one that we have seen in practical applications anywhere. The point of Fixed sampling is that people write their code usually to start by drawing M samples. Once a researcher decides to apply an adaptive method, we believe they might as well start using IBS.
Also note that fixed sampling has constant time cost for each parameter vector tried, but IBS takes more time for worse vectors (so, if anything, IBS is penalized for sampling in bad regions, while Fixed sampling is relatively unaffected). That’s the opposite of what one wants, hence the threshold.

**This should be taken into account when comparing efficiency between these methods.**

As mentioned in a previous answer to Reviewer #1, the early stopping threshold is rarely reached (e.g., 2% of the time for the Psychometric model). A parameter setting (or model) needs to be particularly bad for the log-likelihood to be below chance level. In our numerical validations, the models we are using are the same models used to generate the data (thus there is no model mismatch), and the parameter space spans generally reasonable settings.

Moreover, as mentioned above, Fixed sampling has constant time cost for each parameter vector tried, but IBS takes more time for worse vectors, which are less useful for optimization. The threshold is introduced as a simple method to ameliorate this issue of IBS.

**7) On several occasions the authors invoke properties of their optimizer to explain aspects of the simulation results (e.g., bottom of p.43; see more examples in the minor points below). It's very difficult to understand these considerations without good knowledge of the optimiser (which I am unsure if most readers will have).**

Unfortunately there is no such thing as a "free lunch" in optimization, especially with noisy target functions: any optimizer will have to make fairly strong assumptions to navigate effectively through potentially very noisy function evaluations. For this reason, some details of our results will inevitably depend on the implicit or explicit assumptions and heuristics used by the chosen optimizer.

For the purpose of our paper, we chose an optimizer, BADS, that has been repeatedly shown to perform extremely well with stochastic functions against a whole array of different optimization methods. BADS is becoming the *de facto* state-of-the-art in the field (at least for MATLAB users, as the BADS toolbox exists for now only in MATLAB), with currently 78 citations on Google Scholar and 150 "stars" on GitHub. For more information, see "Practical Bayesian Optimization for Model Fitting with Bayesian Adaptive Direct Search" (Acerbi and Ma, 2017, *NeurIPS*), and the toolbox website: https://github.com/lacerbi/bads

In the revised manuscript, we added a paragraph of explanation that briefly describes how BADS works:

"*BADS works by alternating between two stages: in the Poll stage, the algorithm evaluates points in a random mesh surrounding the current point, in a fairly model-free way; in the Search stage, following the principles of Bayesian optimization (Jones et al., 1998), the algorithm builds a local Gaussian process model of the target function, and chooses the next point by taking into account both mean and variance of the surrogate model, balancing exploration of unknown but promising regions and exploitation of regions known to be high-valued (for maximization).*
*By combining model-free and powerful model-based search, BADS has been shown to be much more effective than alternative optimization methods particularly when dealing with stochastic objective functions, and with a relatively limited budget of a few hundreds to a few thousands function evaluations (Acerbi and Ma, 2017). We refer the interested reader to Acerbi and Ma (2017) and to the extensive online documentation for further information about the algorithm.*"

We believe that a detailed description of the inner workings of this optimizer is beyond the scope of our paper.

**I was even unsure whether their fitting algorithm relies on ML estimation or on some form of Bayesian estimation. Isn't the point here to compare the methods based on ML estimation?**

Our analyses use maximum-likelihood estimation (that, is we optimize the log-likelihood), as stated in the manuscript.

However, any stochastic optimization method makes assumptions, implicitly and explicitly, about the surface being optimized (e.g., smoothness) and about the function noise. Methods which ignore the noise and do not try to deal with it in some way perform extremely poorly, as shown in the extensive benchmark of Acerbi and Ma (2017, *NeurIPS*). Intuitively, a method which treats noisy observations as if they were the true function values will terminate much sooner, sometimes almost immediately, believing that a "spike", due to noise, is a (local) optimum. For this reason, the optimization algorithm needs to somehow (implicitly or explicitly) deal with measurement noise in the target function.

In particular, our chosen optimizer is a hybrid method which combines a (relatively) model-free, "direct-search" part (a variant of *patternsearch*) with a model-based search which performs Gaussian-process based local Bayesian optimization (we refer the reviewer to numerous reviews on Bayesian optimization, such as Shahriari et al., "Taking the human out of the loop: A review of Bayesian optimization", 2015, *Proc. IEEE*).
The "Bayesian" aspect (e.g., priors) induced by the Gaussian process is on the shape and smoothness of the target function. Despite the fact that the inner workings of the optimizer follow some form of Bayesian inference over the function being optimized, the *target function* of the optimization is still the log-likelihood, so we are performing ML estimation.

However, the whole story is more complicated.
When the signal-to-noise ratio in the target function is very small (e.g., in a plateau of likelihood landscape), some of the bias of the optimizer will necessarily play a role in the optimization. Even a seemingly "neutral" algorithmic decision such as how long the optimizer should keep searching over a presumed plateau and when it should stop and return the currently estimated optimum will induce an effective prior in parameter space. Note that this type of algorithmic decision pertains to *any* optimizer, it's not just a bias induced by our chosen optimizer.

While we believe all these issues are important and we strongly believe practitioners should not take optimizers as black-boxes, we are also of the opinion that a detailed analysis and explanation of the interaction between optimizer and target function is beyond the scope and focus of this paper, and distracting from our main points.

**Fig. 9F shows an example where "fixed" seems to fare better than IBS. However, the explanations the authors provide don't make the reasons for that clear and they seem to imply that this is a consequence of the specific way in which the optimiser is used. Is this true? I think this is confusing. If fixed performs better, how can this happen, given all the limitations of the method?**

We suspect that the reviewer is referring to panel 9I (in panel 9F, IBS is better than Fixed).
It is true that by the RMSE metric and for this parameter Fixed is comparable to or even slightly better than IBS (the difference is 2.4 - 3.3% less), but this result should not be too confusing or unexpected.

Our argument throughout the paper is that IBS is *generally* better than fixed sampling in a number of ways (in fact, we dare say almost always), and that, thus, IBS is the best choice *a priori*. In practice, due to non-trivial interactions of the model, data, and optimization algorithm, there might well be scenarios in which IBS and Fixed sampling perform similarly at a specific task (e.g., recovering this specific parameter), or with Fixed slightly better.

In this same scenario, note that IBS performs substantially better than Fixed at recovering the other parameters (for example, the RMSE of $\eta$ is 19-39% lower for IBS than Fixed, and the RMSE of $\xi$ is 4-13% lower for IBS than Fixed). So, overall, IBS still emerges as the best choice (with all the other advantages of IBS, such as the adaptive choice of number of samples, the ability to easily estimate the variance, etc.).

**8) In section 3.1. – can you be clearer about what you mean by "fixing" (section title). This section seems to mix consideration of bias and efficiency and it is not very clear.**

The title of the section is a pun. By "fixing" fixed sampling we meant finding a simple modification that would solve the divergence issue and produce a (nearly) unbiased estimator with other desirable properties, such as efficiency.
Note that we need to consider *both* unbiasedness and efficiency, otherwise the fix would be trivial: take an arbitrarily large number of samples M. For M tending to infinity, fixed sampling is (in the limit) unbiased. However, such a solution is extremely wasteful and undesirable in practice.

**9) Please show the derivation of eq. 15 as I was unsure about it.**

Good point. We added the full derivation of Equation 15 to the Appendix of the revised manuscript (the derivation per se is quite involved and not particularly interesting, primarily a matter of index manipulation).

**10) In the end of section 4.3. I was confused about the variance estimates of eqs 15-16. Eq.15 pertains to noise in the IBS sampling whereas eq. 16 pertains to some posterior variance. It is not clear how the two are related. The authors later show that eq.16 provides a "calibrated" estimator. I am not sure this term is clear. Can you please define it and explain?**

The idea is that e.g. a 95% confidence interval (or credible interval) is *calibrated* if about ~95% of the estimates fall within the interval. By showing that the entire z-scored distribution of estimates is Gaussian and matches very well a unit normal, we are numerically showing that

credible intervals built using our proposed estimator of the variance are calibrated for any practical value of the interval.

We added the following paragraph of explanation for what we mean by “calibrated” in the text:

“*That is, we expect the fraction of estimates within the credible interval $\hat{L}_IBS ± β \sqrt{\hat{V}_IBS} to be (approximately) Φ(β) - Φ(-β), where Φ(x) is the cumulative normal distribution function and β > 0.*”

**Can you also show (in the cases that you can actually calculate eq.15), how eq.16 are distributed relative to the correct (eq.15) values (rather than just show Fig. 3C)?**

For the same datasets and models considered in Fig. 3C, we plot below the conditional probability of obtaining a SD estimate via Eq. 16 (y-axis) given the true SD of the estimator, as per Eq. 15 (x-axis). The estimates using the Bayesian estimator are tightly centered along the diagonal, that is close to the true value.
We believe that this figure is redundant with Fig. 3C, because if the estimates were not tightly distributed along the diagonal, we would see deviations from normality in Fig. 3C, so we did not include it in the text.

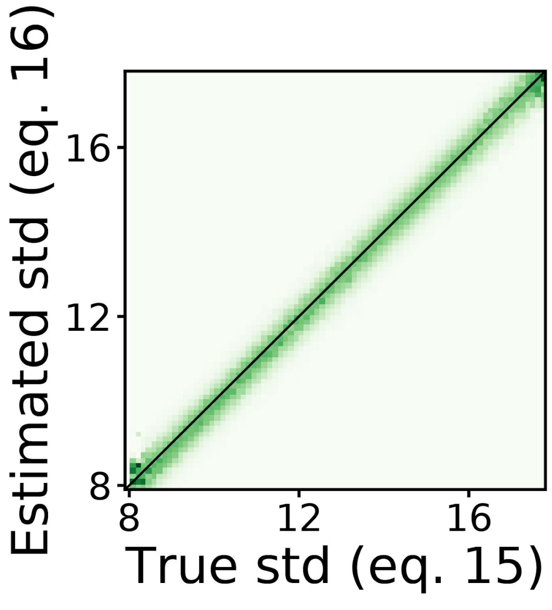


**11) Fig. 7 panel C. How come fixed and IBS perform better than exact?**

Please see the answer in the text, and also previously in response to Rev #1.

**12) For the "early stopping method": I think this method inflates the variance of the log-likelihood estimation because it introduces correlations between log-likelihood estimators on different trials. Is this right? If so please discuss/explain implications.**

This is simply a technique to avoid wasting computations in very bad regions of parameter space (e.g., when the model performs worse than chance level). As also explained in previous answers, we do not expect the early stopping method to have any bearing in regions where the value of the log-likelihood actually matters (unless we are dealing with a terrible model that performs no better than chance, which should nevertheless be discarded via model selection).

**Additionally, it was not very clear when the use of this method is recommended: is this method useful only when searching for ML? What if we're actually interested in obtaining the log-likelihood for different sets of parameters (e.g., to find posteriors)?**

We are recommending this simple fix in combination with maximum-likelihood estimation (or, similarly, maximum-a-posteriori), just to prevent the optimizer from wasting too much time when probing particularly "bad" regions in parameter space.
It is likely that such an approach would also be convenient when performing posterior inference, although one would need to add an additional check that the chosen threshold (e.g., "chance level") differs enough in terms of log-likelihood from the "typical posterior set" in which the posterior distribution lives, otherwise this technique would introduce additional biases (this problem does not arise with optimization). We also note that more sophisticated adaptive thresholds could be used in different parts of parameter space, but developing such methods is beyond the scope of the current paper and left as future work.

More in general, if one wants to infer the log-likelihood for a specific parameter vector, then an early stopping threshold should not be applied.

**Finally- it seems very wasteful to ignore samples of outcomes that were already matched (e.g., the k=5 sample, in Fig. 18). In essence these samples constitute another "run" of IBS so it seems they should be used to improve the variance of the estimator.**

The main idea is that in an efficient low-level implementation (e.g., in C/C++), those samples are never drawn in the first place, thus saving time.
In practice, with modern high-level languages things are more complicated, because one needs to take into account function call overheads, memory limits, the cost of sampling, etc., but a full numerical treatment is beyond the scope of this paper.
Code-savvy and curious readers can go and check under the hood of the numerical open-source implementations that we provide here, which use several tricks for maximal efficiency in terms of runtime speed: https://github.com/lacerbi/ibs
For example, the MATLAB code that we provide exploits what we call "adaptive accelerated vectorization" to automatically maximize performance while balancing several numerical aspects, with no required intervention from the user.

**Minor points**

**1) The last sentence in "conditional simulation" section is unclear. If we can simulate full sequences then we can necessarily produce conditional simulation by using only the full sequences that satisfy a condition – no? I can see that this can costly but this section is not really about costs.**

Yes, one can always perform "rejection sampling", but we wanted to be upfront about limitations. Rejection sampling is not a feasible solution because its cost grows *exponentially* in the number of trials, which quickly enters the realm of physical impossibility.
We modified the sentence accordingly, since this point also puzzled Reviewer #1. Now we say that "*it might not be easy or computationally tractable to generate the response in a given trial, conditional on a specific sequence of previous responses*".

**2) p. 19 first paragraph: "p_i will be 0.5" – I think this is a mistake. It will be close to the lapse rate.**

Yes, it is 0.5 times the lapse rate - thanks for catching this.

**3) Algorithm 1. I think it would be helpful to explain that if algorithm 1 is used within a search in the parameter space then just taking the maximal likelihood during the search is problematic. In fact, in the simulation studies you recalculated the log-likelihood for the best parameters- right?**

Yes, this is a subtle point which is worth stressing, thanks for pointing this out. We clarified it in the text as follows:

"*At the end of optimization, most methods will then return a candidate solution θ* and possibly an estimate of the value of the target function at θ*. However, since the target function is noisy and the final estimate is biased (because, by definition, it is better than the other samples), we recommend to re-estimate \hat{L}_ibs(θ*) multiple times to obtain a higher-precision, unbiased estimate of the log-likelihood at θ* (see also Section 4.4).*"

**4) Fig. 5, the caption refers only to R=1 but R=3 is also used – right? I think similar issues pertain to other figures as well. Please clarify these issues.**

Thanks for noting, we fixed the caption.

To clarify, panels A,D show example values of R (for IBS) and M (fixed sampling), with R chosen such that the average number of samples for IBS would be lower than M; to show that IBS works better than, or as well as, Fixed and with fewers samples. For panel A and panel D we chose different values of R for variety, just to showcase different behaviors.
A full comparison (for all values of tested R and M) is reported in the other panels, in which the x-axis ("number of samples") correspond to different choices of M (and R).

**Also, it can be confusing that the number of samples in A,D are not the same because the two parameters are estimated simultaneously - right?**

The parameters are estimated simultaneously, but we have different runs for different values of R (and M).

**5) I found some of the explanations about the simulation results unclear:**
**a) p.33: "IBS has a slight tendency to underestimate lambda, which is a result of an interaction of the uncertainty handling in BADS with our choice of model parametrization and parameter bounds." This was unclear. Additionally, exact also shows a bias – why?**

This is a well-known problem of maximum-likelihood estimation of lapse parameters, as mentioned in the text, and there are several papers that cover this topic in the psychophysics literature. General approaches to solve issues with lapse estimation include using Bayesian methods, using a very narrow range for the lapse parameter, or measuring the lapse parameter within an ad-hoc condition (see e.g. Prins, 2012, *Journal of Vision*).
As we previously discussed for other details of the optimization, while we think that these are important modeling questions, they do not represent the focus of our paper and we are concerned that spending too long on these issues might distract the reader from our main message.

**b) p.42: "IBS and fixed sampling perform seemingly better here because the interaction between a ridge (or flat region) in the true likelihood landscape and noisy estimates thereof depend on specifics of the problem and of the optimization procedure". Unclear.**

This is the problem we explained above in answer to a previous question about the optimizer.

**Additionally, looking at Fig 16 C, F it seems gamma is estimated quite accurately but eta isn't. How is this consistent with a trade-off explanation provided in the cited paragraph?**

These are not the same datasets, as explained in the text. When studying parameter recovery for eta, we generated datasets with different etas while we fixed gamma to its true value; and vice versa.

**6) Fig 5 (and others). How is the mean number of samples manipulated for IBS? Is this manipulated by the number of repeats or just occurs due to natural variability?**

The number of samples depends on the number of repeats R.
The number of repeats R we used is listed in the Appendix for each model (e.g., R = 1, 2, 3, 5, 10, 15, 20, 35, 50 for the psychometric model).

**Also, does the average number of samples pertain to the whole execution of the optimizer of just to the best fitting parameters?**

We are keeping track during the whole optimization run.

**7) In fig 5B (and similar figures) there is no shaded area. Additionally, wouldn't it be more informative to show SD rather than SE so that we get an impression of estimation errors that can be expected in single studies? Same question for Figure 10.**

Thanks for the suggestion.
In Fig 5B, there is a shaded area, but in this case is so small that it overlaps with the line (it is visible, for example, in Fig 5E). For the bias plots, we preferred to keep showing the SE (standard error) in the revision because the goal of these plots is to show the *unbiasedness* (or not) of the method.
The *estimation error* of each method, instead, is conveyed by Fig 5C (and similar), which plots the RMSE. We agree that in these plots one would want to get a sense of variability across different datasets and runs, so here we switched from SE to SD.

**8) From the description of the 4 in a row simulation (p.40-41) I didn't understand if the model only consider "single-step" moves or deeper trajectories. If the latter is the case it is not clear how these are considered.**

It considers deeper trajectories. A full model specification would take too much space (in fact, please consider that Reviewer #1 has asked us to *reduce* the space used for describing the examples), so we refer the interested reader to the paper in which the model is presented.

**9) Eq. 27: second row: The E (Expectation) seems redundant**

Thanks, good catch.

**10) Caption of Fig. 17: “smaller bias in estimating [ksi]”. The different parameters are on different scales….Do you mean bias measured in %?**

Yes. We intended it as a more qualitative statement.

**11) The explanation below eq. 36 is unclear. First of all: in “exceeds a lower bound” you actually mean falls below a lower bound – right?**

Yes, thanks for catching. In earlier drafts of the article we considered “negative log-likelihoods” (because optimization is implemented as minimization), this is an artifact from those days.

**Second, please explain more clearly why a bias is exponentially small in N.**

The effect of early stopping is to truncate the distribution of log-likelihood estimates obtained by IBS with a threshold. This induces a bias, but the bias will be proportional to the probability of the estimates exceeding the threshold. Since IBS obtains its estimate by summing independent estimators across multiple trials, we can use results from large deviation theory, a field of probability theory concerned about the asymptotic behaviour of remote tails of sequences of probability distributions. One central result in this literature is the Hoeffding inequality [1], which states that sums of independent bounded random variables have exponential (or even lighter) tails. This theorem can be extended to unbounded random variables as well [2].

More intuitively, since the IBS estimator is normally distributed, the influence of the threshold on the estimate is negligible as long as the threshold is several SDs lower than the mean of the estimate. In practice, early stopping should only “kick in” in bad regions of parameter space, and not affect the estimates close to the optimum.

[1] Hoeffding, Wassily. "Probability inequalities for sums of bounded random variables." The Collected Works of Wassily Hoeffding. Springer, New York, NY, 1994. 409-426.
[2] Bentkus, Vidmantas. "An extension of the Hoeffding inequality to unbounded random variables." Lithuanian Mathematical Journal 48.2 (2008): 137-157.

**12) p.77: “divides the budget equally across trials”. Do you mean equal R_i (across trials) or equal R_i/p_i??**

Equal R_i/p_i

**13) Eq.38 considers average sampling cost. What if we consider worse case?**

Thanks for the interesting idea. In theory we could consider higher quantiles or some other metric of “worse case”. However, it is not obvious if we would be able to solve the constrained optimization problem analytically (and a numerical solution could be expensive). It is an interesting question for future work.

**I hope the authors find these thoughts/suggestions helpful for their work**
**Rani Moran.**

Thank you again for the insightful comments!
