# Supplementary material for: Unbiased and efficient log-likelihood estimation with inverse binomial sampling
Source: PLoS Comput Biol. 2020 Dec 23;16(12):e1008483. doi: 10.1371/journal.pcbi.1008483 (PMC7758077; doi:10.1371/journal.pcbi.1008483)
Supplement: Attachment — Submitted filename: ResponseToReviewers2.pdf [file pcbi.1008483.s003.pdf]

Unbiased and Efficient Log-Likelihood Estimation with Inverse Binomial Sampling

Response to reviewers

We thank the reviewers again for the useful feedback on our revised manuscript. In the following, we reply in detail to the few remaining reviewers' comments.

Bas van Opheusden
Luigi Acerbi
Wei Ji Ma

Comments to the Authors:

Reviewer #1: I thank the authors for the careful considerations of my issues in both the response letter and revised article. While I still believe the main points could be made in a much shorter paper, I recognize that my appreciation for brevity and simplicity is not shared by everyone, and this is really an editorial issue, not a scientific one. I'm otherwise happy with the revised manuscript.

Thanks for the comment and for the useful suggestions in the previous review.
We completely agree that brevity is a virtue and a desirable goal in many cases, but unfortunately for a didactic paper like ours it seems like a longer, more verbose format can be useful to the reader.

Reviewer #2: 1. There are some typing errors. For example, Introduction, line 26: "such at" should be "such as". Page 7, line 236: x_n should be x_k.

Thanks for spotting these. We fixed the typo.

2. The authors added the resuls for heavy-tailed distribution, but it is better to add outliers showing robust to outliers

We note that the reviewer, in the previous round, asked "*Also, when the data are from the heavy-tailed distribution, how is the result?*" and this is indeed what we showed with new analyses in our previous response to the reviewers.

With respect to outliers, we remark that an outlier can be defined as a data point which is unlikely under the current model. The three models we considered in the original manuscript already contain "outliers" because their generative model includes a lapse parameter, which causes the model to occasionally respond randomly. On trials where the model's response in the absence of lapses is highly predictable (for example, in the orientation discrimination task with a stimulus far from the decision boundary), lapse responses are outliers.

The lapse parameter of a model is also exactly the component that deals with outliers during parameter estimation. As mentioned in the paper, we strongly recommend adding a lapse rate to the models one fits using IBS, to ensure that all observations have a non-negligible probability of being sampled.
Finally, we note that, regardless of the generative model and what "outliers" it contains, IBS will compute an unbiased estimate of the log-likelihood for *any* dataset, as long as the model satisfies the assumptions listed in the paper. Therefore, there is nothing to be proven besides what already is in the manuscript — at worst, it will take longer to sample an unlikely observation (hence the lapse rate mentioned above, or tricks such as the early-stopping suggested in the Appendix).

In summary, we think our analyses already corroborate that IBS (when combined with BADS) is able to work in many real-world cases, including datasets with "outliers" (i.e., low-probability observations).

Reviewer #3: The authors provided an excellent revision. There is one remaining issue, I believe the authors should clarify, and I have a few minor suggestions to improve clarity.

Thanks for the additional comments.

1) In L. 760-763. But in the absence of the 'exact' likelihood-form we will also not have access to the true ML – right? If I get this correctly, the exact method allowed you to find the "true" ML, but without the exact method, how can we know the true ML and how can we calculate the likelihood loss?

This is correct, in this figure we are using the exact log-likelihood calculation to quantify the errors made by approximate methods. The log-likelihood loss is indeed not something a *researcher* would compute lacking ground truth, instead it is a quantity that we use in the paper to demonstrate how close the method gets to ground truth in two of the considered scenarios.

Note that this is similar to our analyses of parameter estimates, where we compare fixed sampling and IBS to exact maximum-likelihood estimation. The logic is the same. What the reviewer is currently asking is akin to asking how could the user compute the bias without knowing the true parameter values — indeed, they cannot!

We believe the text is clear in saying: "*In the previous sections, we have analyzed the bias and error of different estimation methods when recovering the generating model parameters in various scenarios. Another important question, crucial for model selection, is how well different methods are able to recover the true maximum log-likelihood*."

2) I still think that the explanations that involve properties of the optimizer are unclear and simply compromise the flow of the writing and distract readers (e.g., L 663-668). I appreciate this is not the main point of the paper. Perhaps the authors can delegate these

issues to a supplementary section and provide some more details for interested readers there (e.g., I found some responses in the rebuttal letter clearer than these short explanations in the main text).

We reduced the explanation in lines 663-668, referring the reader to other material about, e.g., the problem of parameter identifiability and the shape of likelihood landscapes. We agree that disentangling properties of optimization algorithms and inductive biases inherent in their structure is fascinating, however we believe that a detailed discussion of this point is beyond the scope of the current manuscript. We also note that other reviewers have urged towards brevity. Unfortunately, we cannot do justice to this topic without adding considerable content to the main text or Supplement, content which would be tangential to the focus of the manuscript. It is a complex topic, and perhaps we will write a separate tutorial on it.

3) I think it will be helpful to clarify to readers in the main text that in the result figures (e.g., Fig 5B) the "number of samples" for IBIS was manipulated via changing the numbers of repeats. Otherwise it is unclear how this happens.

In the "Procedure" section we added: "*In each scenario, we directly compare the two methods in terms of number of samples by computing the* average *number of samples used by IBS for a given number of repeats R.*"

4) L. 579-580: The theoretical and numerical reasons are not really explained to readers.

No, for reasons of space, and it is irrelevant for the points made in the paper.
In short, for the numerical/practical reasons, one would again need to refer to properties of optimization (which is clearly not invariant to reparameterization), and for the theoretical reasons we would need to talk about parameters of unknown scale and Jeffreys priors. Both topics are beyond the scope of this paper.

5) L. 601 "remains impossible". Please be more accurate. Estimation is always possible but may have terrible qualities.

True. We changed "*estimating [the lapse parameter] remains impossible*" with "*estimating [the lapse parameter] accurately remains unattainable*".

6) EQ. 23. The first 'd' in the integral seems spurious?

Thanks for catching the typo. Fixed.

I hope the authors find these comments helpful,
Rani Moran.

Thanks again for the useful comments!
